# Genome-wide analysis of the P450 gene family in tea plant (*Camellia sinensis*) reveals functional diversity in abiotic stress

**DOI:** 10.1186/s12864-023-09619-4

**Published:** 2023-09-11

**Authors:** Chuan Shen, Xia Li

**Affiliations:** 1 Shaannan Eco-Economy Research Center, Ankang University, Ankang, 725000 China; 2 Department of Electronic and Information Engineering, Ankang University, Ankang, 725000 China

**Keywords:** *Camellia sinensis*, P450 gene family, Promoter, Synteny analysis, Gene expression

## Abstract

**Background:**

Cytochrome P450 (Cytochrome P450s) genes are involved in the catalysis of various reactions, including growth, development, and secondary metabolite biosynthetic pathways. However, little is known about the characteristics and functions of the P450 gene family in *Camellia sinensis* (*C. sinensis*).

**Results:**

To reveal the mechanisms of tea plant P450s coping with abiotic stresses, analyses of the tea plant P450 gene family were conducted using bioinformatics-based methods. In total, 273 putative P450 genes were identified from the genome database of *C. sinensis*. The results showed that P450s were well-balanced across the chromosomes I to XV of entire genome, with amino acid lengths of 268–612 aa, molecular weights of 30.95–68.5 kDa, and isoelectric points of 4.93–10.17. Phylogenetic analysis divided CsP450s into 34 subfamilies, of which CYP71 was the most abundant. The predicted subcellular localization results showed that P450 was distributed in a variety of organelles, with chloroplasts, plasma membrane,,and cytoplasm localized more frequently. The promoter region of CsP450s contained various cis-acting elements related to phytohormones and stress responses. In addition, ten conserved motifs (Motif1-Motif10) were identified in the CsP450 family proteins, with 27 genes lacking introns and only one exon. The results of genome large segment duplication showed that there were 37 pairs of genes with tandem duplication. Interaction network analysis showed that CsP450 could interact with multiple types of target genes, and there are protein interactions within the family. Tissue expression analysis showed that P450 was highly expressed in roots and stems. Moreover, qPCR analysis of the relative expression level of the gene under drought and cold stress correlated with the sequencing results.

**Conclusions:**

This study lays the foundation for resolving the classification and functional study of P450 family genes and provides a reference for the molecular breeding of *C. sinensis*.

**Supplementary Information:**

The online version contains supplementary material available at 10.1186/s12864-023-09619-4.

## Background

Cytochrome P450s (CYPs) are the largest enzyme family involved in NADPH- and/or O2-dependent hydroxylation reactions, which are ubiquitous across all domains of life [1]. P450 enzymes are present in all plant species, and play important roles in plant growth, development, and adaptation to the environment [2]. Under terrestrial environments, the preserved P450 families support chemical defence mechanisms, and a number of them participate in the manufacture and catabolism of hormones [3]. Furthermore, through boosting the action of substances (such as flavonoids) with a higher antioxidant activity, CYPs are also implicated in safeguarding plants from harsh environmental circumstances [4, 5]. For the biosynthesis pathways of species-specific metabolites, species-specific P450 families are necessary [6]. All cytochrome enzymes will have the code "CYP" followed by the family number, then an alphabet that designates the subfamily of the enzyme [7]. Their amino acid sequences are extremely diverse, with similarities as low as 16% in some cases, but their structural folding has remained conserved throughout evolution [8].

With the development of next-generation sequencing technology (NGS), a large number of plant genomes have been published, which has also facilitated the identification of gene families [9]. As one of the largest gene superfamily in plant genomes, P450s are represented by more than 300,000 gene sequences that have so far been preserved in databases, which include more than 16,000 plant P450s [10]. Nonetheless, the identification of P450 gene family members presents a significant challenge due to their vast quantity, comprising no less than 1% of the total annotated genes in plant genomes. Consequently, this results in a comparatively lower number of identified P450 gene families. Research has shown that *Arabidopsis thaliana* (*A. thaliana*) has 246 P450 genes, making it the third-largest gene family in *A. thaliana* [11]. The number of P450 genes in other plants is also relatively high, such as 457 in grape (*Vitis vinifera*), 332 in soybean (*Glycine max*), 312 in poplar (*Populus trichocarpa*), 356 in rice (*Oryza sativa*), 372 in sorghum (*Sorghum bicolor*) [12], 233 in tomato (*Solanum lycopersicum*) [13], 174 in mulberry (*Morus notabilis*) [14], 334 in flax (*Linum usitatissimum* L.) [15], 263 in tobacco (*Nicotiana tabacum*) [16], and 258 in Chinese cabbage (*Brassica rapa* L.) [17]. Therefore, whole-genome analysis and co-expression networks of P450 gene families can help to determine the functions of P450s and understand the evolution of these multifunctional enzymes.

P450 enzymes are classified into different subfamilies based on their amino acid sequence and function. Plant P450s have been shown to participate in various biochemical pathways to produce primary and secondary metabolites, such as phenylpropanoids, alkaloids, terpenoids, lipids, cyanoglycosides, and polyols, as well as plant hormones [18]. For example, gene families *CYP90*, *CYP724*, and *CYP734* are involved in the biosynthesis of steroidal saponins and sugar alkaloids [19]. P450 enzymes can also participate in the regulation of plant growth and development by synthesizing hormones [20], such as CYP735As involved in the biosynthesis of cytokinins [21], CYP707A involved in the catalytic synthesis of abscisic acid [22], CYP701A, CYP88AC, CYP714A1, CYP714D1, and CYP714A2 involved in the synthesis and inactivation of gibberellins [23, 24], CYP85A, CYP90A, CYP90B, CYP90C, CYP90D, CYP724B, and CYP734A involved in the biosynthesis of brassinosteroids [25–27], and CYP74A, CYP94B3, CYP94C1, CYP74A, and CYP74B involved in the synthesis of jasmonic acid [28–30].

P450 enzymes have also been shown to play a role in plant stress responses, including responses to abiotic stress (such as drought and extreme temperatures) and biotic stress (such as insect and pathogen attacks) [31, 32]. For instance, after *Xanthomonas axonopodis* infection, the CYP gene CaCYP1 from *Capsicum annuum* was discovered to be implicated in the (hypersensitivity response) [33]. It was discovered that the *Arabidopsis* CYP gene, *AtCYP76C2*, is linked to hypersensitive fast cell death, a defensive mechanism against bacterial canker (*Pseudomonas syringae*) infection [34]. Such CYP genes are excellent candidates to be exploited in agricultural species engineering to make them resistant to biotic and abiotic stress. Besides, P450 genes have been found to be involved in the metabolism of heavy metal stress [35]. Overall, the P450 gene family plays a key role in the metabolism of various compounds in plants, and understanding the functions of these enzymes is important for studying plant biology and developing new plant-derived products.

Tea (*Camellia sinensis*) is one of the most important beverage crops in the world, with significant economic and health benefits. With the publication of the tea genome, over 80 tea gene families have been identified, such as HDAC [36], PMF [37], PLD [38], MAPK [39], as well as transcription factor families NAC, bZIP, TCP, and MYB [40–43]. However, few P450 genes from tea have been reported and functionally annotated. Moreover, to date, there have been no reports on the whole-genome study of these genes. Therefore, in this study, we identified the members of the P450 gene family in the whole genome of tea using bioinformatics methods, grouped P450 genes with important functions, and analyzed the physicochemical information, structural function, and expression patterns of all members to understand the molecular evolution of P450 genes and provide a reference for functional characterization of important candidate genes. Furthermore, this investigation holds significant implications for the genetic enhancement of tea growth, development, yield, and resistance to pests and diseases through the utilization of this gene family.

## Materials and methods

### Identification of P450 genes in tea plant genome

In this study, we aimed to identify and characterize P450 genes in the tea plant (*C. sinensis*) genome. To achieve this, we downloaded the HMM (Hidden Markov Model) file for the typical conserved domain of P450 genes (PF00067) from the Pfam 35.0 protein family database (http://pfam.xfam.org). We then used the HMMER3.0 software to perform a comparative search of all protein sequences in the tea plant genome database (http://tpia.teaplant.org).

To increase the accuracy of our search, we obtained 238 AtP450 protein sequences from the TAIR website (https://www.arabidopsis.org/) and used them as queries to perform a local BLAST search in the tea plant genome database (with an E-value cutoff of 10^–3^). We then filtered the candidate protein sequences with incomplete structures using the NCBI-CDD (http://www.Ncbi.Nlm.Nih.Gov/Structure/cdd/wrpsb.cgi) and SMART (http://smart.embl-heidelberg.de/) domain detection tools, resulting in the identification of CsP450 protein sequences.

To further characterize the identified CsP450 protein sequences, we submitted them to the ProtParam (http://web.expasy.org/protparam/) and predicted their molecular weight, isoelectric point, and amino acid composition [44]. Finally, we used TBtools (https://github.com/CJChen/TBtools/releases) to locate the *CsP450* genes on the tea plant chromosomes and named them according to their positions on the chromosomes [45].

### Phylogenetic analysis of CsP450s

To identify the gene family members, protein sequences were extracted based on their IDs and aligned with 238 family genes from *A. thaliana* using Clustal W software with the default parameters [46]. The resulting alignment was used to construct an unrooted evolutionary tree using the Neighbor-Joining method using MEGA 7 software (https://www.megasoftware.net/) [47]. The Bootstrap parameter was set to 1000 to ensure the robustness of the tree. The resulting tree was further annotated using EvolView (https://www.evolgenius.info/evolview/#login) to enhance its readability and visual presentation.

### Analysis of CsP450s gene structure and cis-acting elements

In this study, the CDS and genomic annotation information of the CsP450 gene family was obtained from the tea plant genome database. The Gene Structure Display Server (GSDS, http://gsds.cbi.pku.edu.cn/) was used to generate a schematic representation of the gene family's exon–intron structure [48]. The MEME online software (https://meme-suite.org/meme/) was used to analyze the conserved motifs of the CsP450 proteins, with the following parameters: maximum of 10 misfits and an optimum motif width of 6—200 amino acid residues [49]. The gene family's evolutionary tree, gene structure, and motif analysis were combined in a single figure using the TBtools software to demonstrate the gene structure and evolutionary relationships between family members.

To further explore the regulatory elements of the CsP450 gene family, the 2 kb upstream region of the ATG start codon of the CsP450 genes was downloaded from the tea plant database. The PlantCARE online tool (http://bioinformatics.psb.ugent.be/webtools/plantcare/html/) was used to predict cis-acting elements in the promoter sequences [50], and the results were visualized using TBtools.

### Subcellular localization prediction of CsP450s gene

WOLF PSORT ProtParam tool (https://wolfpsort.hgc.jp/) were used to predict the subcellular localization of CsP450-encoded proteins. The algorithm of WOLF PSORT ProtParam tool compares the input sequence to the database of known subcellular localization signals and motifs, and then assigns a probability score to each potential subcellular localization site.

### Chromosomal localization and genome collinearity analysis of CsP450s gene

To perform chromosome localization analysis of the gene family, we used the software MapChart (https://academic.oup.com/jhered/article/93/1/77/2187477). We conducted genome-wide collinearity analysis and gene duplication event analysis using the software McscanX with default parameters [51]. KaKs Calculator 2.0 was used to estimate the non-synonymous substitution rate (Ka), synonymous substitution rate (Ks), and the ratio (= Ka/Ks) of paralog pairs for each pair of paralogs [52]. In general, Ka/Ks = 1 reflects neutral selection (pseudogenes), Ka/Ks =  < 1 shows purifying or negative selection, and Ka/Ks =  > 1 shows positive selection.

### Protein–protein interaction network analysis of CsP450s

The candidate P450 genes of tea plant were not found in the String database (https://string-db.org/). Therefore, we used OrthoVenn2 (https://orthovenn2.bioinfotoolkits.net/home) to search for homologous genes of tea plant P450 genes in Arabidopsis for further analysis. The protein–protein interaction network was visualized using Cytoscape (https://cytoscape.org/) network visualization software, where nodes represented proteins and edges represented interactions..

### In-silico gene expression analysis of CsP450 genes

The Illumina RNA-sequencing (RNA-seq) data of tea plant were downloaded from the tea plant genome database (http://tpdb.shengxin.ren/) to examine the relative expression patterns of *CsP450s* under abiotic stress with various time points (0 h, 24 h, 48 h, and 72 h for PEG) and (0 h, 6 h, and 7 d for cold (4℃)) and different tissues including apical buds, flowers, fruits, young leaves, mature leaves, old leaves, roots, and stems. The clustering heatmap was drawn using the heatmap tool by Biotech Cloud Platform (https://cloud.oebiotech.cn/task/detail/heatmap/), with the parameter settings for clustering rows and selecting FPKM as the data preprocessing method.

### qPCR analysis

Drought stress was induced in tea plants by treating them with 20% PEG6000 for 24 h, 48 h, and 72 h, while the control sample was collected at 0 h. To investigate the response of *CsP450* genes to drought stress, ten CsP450 genes were selected and their expression levels were analyzed using qPCR. Total RNA was extracted from the tea plant samples using the RNAprep Pure Plant Kit (Tianjin, China), and cDNA was synthesized using the PrimeScript® RT reagent kit (Takara, China) according to the manufacturer's instructions. Gene-specific primers were designed using the NCBI database online toolkit (https://www.ncbi.nlm.nih.gov/tools/primer-blast/) and used to amplify the target fragments. The relative expression levels of the selected genes were calculated using the 2^−ΔΔCt^ method [53]. Additionally, cold stress was imposed on the tea plants by treating them at 4℃ for 6 h and 7 d, with samples collected at 0 h as the control. The expression analysis of *CsP450* genes was performed with three biological replicates and three technical replicates for all samples.

### Data analysis

The statistical analysis was performed using IBM SPSS Statistics 22 software (IBM, New York, USA) to compare the differences between treatments. All values presented in the figures are expressed as the mean ± standard deviation (SD) of biological triplicates, unless otherwise stated. Two-way analysis of variance (ANOVA) was conducted to determine the least significant difference (LSD) with a significance level of *p* < 0.05.

## Results

### Identification and physicochemical analysis of CsP450 gene family

After screening the tea plant genome using NCBI-CDD and SMART, 273 candidate P450 genes were identified, and were subsequently designated as *CsP4501* to *CsP450273* according to their chromosome location, numbering and naming (Table 1). The chromosomal distribution of the P450 genes was found to well-balanced, with genes located on chromosomes 1 to 15. The P450 protein sequences varied greatly in length, ranging from 268 to 612 amino acids, with molecular weights ranging from 30.95 to 68.5 kDa, and isoelectric points ranging from 4.93 to 10.17. Subcellular localization analysis showed that these proteins were mainly localized to organelles such as chloroplasts, plasma membranes, cytoplasm, endoplasmic reticulum, mitochondria, nuclei, and vacuoles.

**Table 1:** The physiological and biochemical properties of 273 CsP450 proteins in *C. sinensis*. Plas: plasma membrane; E.R.: endoplasmic reticulum; Mito: mitochondria; Chlo: chloroplast; Extr: extracellular; Cyto: cytoplasm; vacu: vacuole; nucl: nucleus; golg: Golgi apparatus; pero: peroxisome, cysk: cytoskeleton

Gene name	Gene ID	Chromosome	Start site	End site	Length/AA	MW(Da)	pI	Subcellular localization
CsP4501	CSS0002756	Chr1	16,745,874	16,747,688	512	57,785.6	9.33	chlo: 6, plas: 4, cyto: 3
CsP4502	CSS0031915	Chr1	16,921,255	16,923,103	512	57,785.6	9.33	chlo: 6, plas: 4, cyto: 3
CsP4503	CSS0048419	Chr1	37,553,534	37,572,711	437	50,010.5	7.09	extr: 4, chlo: 3, vacu: 2
CsP4504	CSS0008024	Chr1	40,243,989	40,255,785	429	48,945.9	7.74	chlo: 11
CsP4505	CSS0016245	Chr1	48,668,112	48,676,336	510	57,191.9	9.75	nucl: 3.5, chlo: 3, cysk_nucl: 2.5
CsP4506	CSS0004602	Chr1	50,059,069	50,063,569	406	45,091.1	8.48	chlo: 4, golg: 4, cyto: 2, plas: 2
CsP4507	CSS0018227	Chr1	118,908,868	118,910,932	556	63,280.6	7.2	E.R.: 3, chlo: 2, nucl: 2, cyto: 2
CsP4508	CSS0028563	Chr1	151,751,669	151,754,561	503	57,569.4	7.72	chlo: 6, vacu: 3
CsP4509	CSS0047168	Chr1	153,388,574	153,390,278	536	59,717.2	7.76	chlo: 4, nucl: 3, plas: 3, E.R.: 2
CsP45010	CSS0041122	Chr1	153,752,301	153,754,005	536	59,660.2	7.48	chlo: 4, nucl: 3, plas: 3, E.R.: 2
CsP45011	CSS0031581	Chr1	153,945,493	153,947,001	502	57,693.2	8.62	chlo: 10
CsP45012	CSS0039570	Chr1	154,508,151	154,509,748	500	57,802.7	8.68	cyto: 4, E.R.: 3, vacu: 2
CsP45013	CSS0014246	Chr1	154,577,044	154,581,121	500	58,102.5	9.52	cyto: 5, E.R.: 4, chlo: 2
CsP45014	CSS0041298	Chr1	154,906,481	154,908,279	502	57,818.3	7.92	chlo: 11
CsP45015	CSS0031367	Chr1	155,037,372	155,038,882	492	56,474.6	7.33	chlo: 11
CsP45016	CSS0000604	Chr1	155,431,461	155,435,423	462	53,825.4	9.94	chlo: 6.5, chlo_mito: 4.33, cyto: 4
CsP45017	CSS0001248	Chr1	155,539,753	155,541,261	502	57,525	7.69	E.R.: 6, plas: 2
CsP45018	CSS0021984	Chr1	155,696,666	155,698,206	509	58,818.8	6.7	E.R.: 6, cyto: 5
CsP45019	CSS0016008	Chr1	174,716,121	174,723,142	515	58,188.6	6.11	chlo: 4, plas: 3, cyto: 2
CsP45020	CSS0037386	Chr1	190,380,278	190,383,548	501	56,927.3	7.37	chlo: 12
CsP45021	CSS0004078	Chr1	190,458,787	190,468,201	432	49,238.9	9.31	chlo: 12
CsP45022	CSS0050435	Chr1	194,039,136	194,041,670	406	46,700.6	9.61	chlo: 10, cyto: 2
CsP45023	CSS0009881	Chr1	201,238,701	201,245,001	517	59,173.9	9.46	vacu: 8, mito: 2
CsP45024	CSS0009174	Chr1	204,553,198	204,557,012	465	53,622.1	8.92	chlo: 7, nucl: 3.5, cysk_nucl: 2.5
CsP45025	CSS0045183	Chr2	4,546,183	4,549,597	478	54,132.4	8.8	chlo: 10, nucl: 2
CsP45026	CSS0004050	Chr2	15,736,618	15,738,937	374	43,241.4	7.64	chlo: 9, cyto: 2, mito: 2
CsP45027	CSS0014427	Chr2	15,840,686	15,843,006	374	43,158.2	7.34	chlo: 10, cyto: 2
CsP45028	CSS0032837	Chr2	18,990,351	18,993,526	516	58,820.2	9.01	chlo: 6, extr: 4, cyto: 2, vacu: 2
CsP45029	CSS0024578	Chr2	30,971,834	30,976,761	488	55,495.5	8.45	chlo: 12
CsP45030	CSS0008808	Chr2	31,864,544	31,875,008	497	56,325.5	7.73	plas: 5, chlo: 4, E.R.: 4
CsP45031	CSS0033945	Chr2	34,499,848	34,539,529	435	49,436	9.43	chlo: 4, nucl: 2.5, cyto: 2, extr: 2
CsP45032	CSS0013355	Chr2	36,174,414	36,177,201	498	55,797.6	8.14	plas: 8, E.R.: 4
CsP45033	CSS0007433	Chr2	36,256,210	36,259,051	498	55,741.5	7.87	plas: 8, E.R.: 4
CsP45034	CSS0038003	Chr2	36,352,656	36,359,616	497	56,113.5	6.05	chlo: 9, cyto: 2
CsP45035	CSS0020543	Chr2	40,829,062	40,837,806	492	56,176.5	7.13	chlo: 4, nucl: 3, cyto: 2, extr: 2
CsP45036	CSS0000373	Chr2	42,922,610	42,927,076	512	58,021.3	6.16	chlo: 11
CsP45037	CSS0028399	Chr2	73,633,278	73,635,425	526	58,160.9	6.58	chlo: 10, nucl: 2
CsP45038	CSS0049181	Chr2	73,943,547	73,945,693	526	58,176.8	6.45	chlo: 10, nucl: 2
CsP45039	CSS0049143	Chr2	87,889,230	87,892,861	512	57,837.4	7.68	chlo: 12
CsP45040	CSS0032768	Chr2	87,958,780	87,965,419	486	55,021.2	7.39	chlo: 11
CsP45041	CSS0036137	Chr2	88,014,483	88,019,993	486	55,016.2	7.2	chlo: 11
CsP45042	CSS0003805	Chr2	88,426,283	88,428,989	509	57,883.9	6.87	chlo: 10, extr: 2
CsP45043	CSS0031608	Chr2	89,673,725	89,676,697	509	57,859.8	6.8	chlo: 9, extr: 2
CsP45044	CSS0048024	Chr2	90,322,826	90,326,296	512	57,815.4	7.44	chlo: 11
CsP45045	CSS0028343	Chr2	90,802,332	90,809,318	512	58,023.6	7.41	chlo: 9, extr: 2
CsP45046	CSS0042891	Chr2	90,880,290	90,891,404	486	55,063.3	6.89	chlo: 11
CsP45047	CSS0000824	Chr2	113,487,930	113,496,798	519	59,866.8	9.95	plas: 8, E.R.: 3
CsP45048	CSS0003621	Chr2	145,498,151	145,500,109	542	60,965.2	9.31	chlo: 5, mito: 4, nucl: 2
CsP45049	CSS0040835	Chr2	208,984,450	208,987,864	509	58,235.2	8.4	chlo: 9, nucl: 2
CsP45050	CSS0003650	Chr3	7,869,027	7,874,443	496	55,557.5	6.39	chlo: 12
CsP45051	CSS0004890	Chr3	16,392,350	16,396,880	474	54,691.2	9.22	chlo: 4, nucl: 3.5, cysk_nucl: 2.5
CsP45052	CSS0031469	Chr3	129,101,192	129,110,875	474	54,490.7	8.62	plas: 7.5, golg_plas: 5, E.R.: 3
CsP45053	CSS0017781	Chr3	130,026,062	130,036,339	474	54,469.6	8.23	plas: 7, E.R.: 3
CsP45054	CSS0050358	Chr3	161,112,555	161,117,094	518	59,921.5	8.62	chlo: 4, vacu: 3, nucl: 2.5
CsP45055	CSS0025742	Chr3	161,643,496	161,648,033	516	59,607	8.34	chlo: 5, vacu: 3, nucl: 2.5
CsP45056	CSS0002167	Chr3	161,804,333	161,811,518	516	59,725.2	8.49	chlo: 4, vacu: 3, nucl: 2.5
CsP45057	CSS0002726	Chr3	161,906,506	161,911,856	508	57,933.4	9.21	chlo: 13
CsP45058	CSS0029260	Chr3	161,921,295	161,925,877	511	58,518.9	8.49	chlo: 13
CsP45059	CSS0004642	Chr3	163,183,576	163,186,467	499	55,981.8	7.5	chlo: 9, plas: 2
CsP45060	CSS0012719	Chr3	163,264,929	163,270,773	424	47,735.1	8.23	chlo: 9, cyto: 5
CsP45061	CSS0009410	Chr3	163,315,501	163,323,845	499	55,938.8	7.68	chlo: 6, nucl: 2, cyto: 2, mito: 2
CsP45062	CSS0043978	Chr3	163,343,627	163,346,633	499	55,926.8	7.17	chlo: 11
CsP45063	CSS0027589	Chr3	163,430,946	163,436,993	480	53,792.3	8.47	chlo: 9, cyto: 2
CsP45064	CSS0003998	Chr3	163,516,436	163,519,058	495	55,458.9	8.57	chlo: 8, nucl: 2
CsP45065	CSS0050026	Chr3	183,463,703	183,466,216	535	60,254.4	8.55	chlo: 6, nucl: 2, cyto: 2
CsP45066	CSS0019385	Chr4	9,466,713	9,471,635	472	53,482.4	7.13	chlo: 7, nucl: 2
CsP45067	CSS0000506	Chr4	9,630,165	9,631,936	494	56,321	7.76	chlo: 12
CsP45068	CSS0002199	Chr4	11,780,285	11,787,442	501	57,563.1	8.2	vacu: 5, plas: 2, extr: 2
CsP45069	CSS0025787	Chr4	16,895,745	16,899,347	512	57,727.2	7.95	chlo: 13
CsP45070	CSS0039798	Chr4	19,160,723	19,164,347	471	53,614.9	10.01	chlo: 10, cyto: 2
CsP45071	CSS0027354	Chr4	20,182,445	20,186,376	471	53,642.9	10.02	chlo: 10, cyto: 2
CsP45072	CSS0034616	Chr4	21,930,880	21,933,141	514	58,130.3	7.16	plas: 7, E.R.: 6
CsP45073	CSS0005190	Chr4	21,945,620	21,950,835	493	55,588.9	8.66	plas: 7, E.R.: 6
CsP45074	CSS0031003	Chr4	22,013,450	22,015,553	495	56,143.5	8.74	chlo: 12, nucl: 2
CsP45075	CSS0013712	Chr4	22,147,136	22,149,142	491	55,199.5	8.83	plas: 7, E.R.: 6
CsP45076	CSS0002018	Chr4	22,379,882	22,387,334	506	57,293.5	8.8	chlo: 12, extr: 2
CsP45077	CSS0036767	Chr4	37,410,540	37,412,827	552	61,531.6	7.15	chlo: 4, plas: 3, nucl: 2.5
CsP45078	CSS0007277	Chr4	67,888,493	67,890,903	512	57,599.4	9.27	vacu: 4, plas: 3, E.R.: 3
CsP45079	CSS0014497	Chr4	68,316,175	68,318,216	512	57,516.4	9.17	plas: 5, vacu: 3, E.R.: 3
CsP45080	CSS0026934	Chr4	68,337,187	68,339,584	512	57,430.3	9.31	plas: 5, vacu: 3, E.R.: 3
CsP45081	CSS0028617	Chr4	68,409,115	68,411,165	517	58,195.3	9.33	plas: 4, vacu: 4, E.R.: 3
CsP45082	CSS0021134	Chr4	104,168,580	104,170,781	517	58,134.2	9.11	plas: 7, E.R.: 4, nucl: 2
CsP45083	CSS0040945	Chr4	104,433,732	104,435,864	462	52,167	8.88	cyto: 5, E.R.: 4, chlo: 2
CsP45084	CSS0010654	Chr4	104,686,173	104,688,382	507	57,366.1	8.37	chlo: 4, nucl: 3.5, cysk_nucl: 2.5
CsP45085	CSS0037742	Chr4	118,447,294	118,457,074	518	58,500.3	8.29	chlo: 9, cyto: 2, E.R.: 2
CsP45086	CSS0029120	Chr4	118,522,810	118,527,185	535	60,066.9	7.72	chlo: 7, extr: 2, vacu: 2
CsP45087	CSS0029403	Chr4	121,944,567	121,947,915	498	55,629.3	7.82	plas: 7, E.R.: 4
CsP45088	CSS0033265	Chr4	122,170,488	122,173,230	495	55,278.9	6.99	plas: 6, E.R.: 4, vacu: 2
CsP45089	CSS0002737	Chr4	128,630,369	128,641,902	505	58,037	9.7	plas: 9, E.R.: 3
CsP45090	CSS0033266	Chr4	154,979,843	154,989,545	529	59,872.7	8.61	chlo: 9
CsP45091	CSS0021647	Chr4	155,905,987	155,924,297	335	37,946.4	6.87	cyto: 6, mito: 3, nucl: 2
CsP45092	CSS0013676	Chr4	163,405,873	163,408,066	485	55,346.5	8.39	chlo: 10, cyto: 4
CsP45093	CSS0013294	Chr5	11,101,662	11,103,768	527	59,849.9	9.17	chlo: 11, cyto: 2
CsP45094	CSS0013535	Chr5	11,131,037	11,132,412	297	34,013.8	5.04	cyto: 9, chlo: 3
CsP45095	CSS0012042	Chr5	17,023,723	17,030,440	510	57,819.4	8.73	chlo: 9, cyto: 2, E.R.: 2
CsP45096	CSS0046278	Chr5	33,226,164	33,231,898	470	53,543.6	8.4	chlo: 9, extr: 2
CsP45097	CSS0026669	Chr5	36,234,259	36,237,306	513	58,205.8	7.7	vacu: 4, E.R.: 4, plas: 3
CsP45098	CSS0040850	Chr5	36,605,215	36,608,011	513	58,261.8	7.52	chlo: 9, extr: 3
CsP45099	CSS0045707	Chr5	36,843,497	36,848,206	510	57,925.4	7.14	chlo: 8, nucl: 2, vacu: 2
CsP450100	CSS0020861	Chr5	47,393,652	47,398,831	432	48,940.3	7.31	E.R.: 4, vacu: 3, plas: 2
CsP450101	CSS0011090	Chr5	47,770,120	47,780,603	443	50,662.4	7.64	vacu: 5, E.R.: 3, plas: 2, golg: 2
CsP450102	CSS0023986	Chr5	91,253,885	91,255,855	512	57,564.3	9.16	vacu: 4, plas: 3, E.R.: 3, chlo: 2
CsP450103	CSS0028297	Chr5	118,948,293	118,983,537	596	67,019.2	7.96	chlo: 11, nucl: 2
CsP450104	CSS0022319	Chr5	119,785,891	119,835,798	590	66,247.3	7.96	chlo: 12
CsP450105	CSS0021097	Chr5	136,256,537	136,259,146	484	54,383.9	8.89	chlo: 7, vacu: 3
CsP450106	CSS0000264	Chr5	136,834,443	136,836,641	484	54,565	8.43	chlo: 7, vacu: 3
CsP450107	CSS0003855	Chr6	1,513,766	1,517,112	489	55,471.7	8.86	chlo: 4, nucl: 2.5, cyto: 2, extr: 2
CsP450108	CSS0016815	Chr6	14,413,367	14,416,421	520	59,495.2	8.5	chlo: 4, plas: 3, nucl: 2.5, cyto: 2
CsP450109	CSS0035549	Chr6	18,242,145	18,244,547	509	58,430.4	8.55	chlo: 6, extr: 3, vacu: 2
CsP450110	CSS0048406	Chr6	18,248,413	18,252,737	513	58,620.8	9.61	chlo: 9, cyto: 2
CsP450111	CSS0036771	Chr6	43,911,647	43,914,478	467	53,483.6	9.67	chlo: 9, nucl: 2, vacu: 2
CsP450112	CSS0010061	Chr6	43,915,261	43,918,093	495	56,273.7	8.53	chlo: 10, vacu: 2
CsP450113	CSS0027259	Chr6	61,820,620	61,824,382	527	60,052.9	9.92	chlo: 4, nucl: 2.5, cyto: 2, extr: 2
CsP450114	CSS0047793	Chr6	61,894,681	61,898,447	527	60,052.9	9.92	chlo: 4, nucl: 2.5, cyto: 2, extr: 2
CsP450115	CSS0037356	Chr6	79,942,440	79,945,010	546	61,595.2	9.1	chlo: 4, cyto: 3, nucl: 2.5, plas: 2
CsP450116	CSS0025111	Chr6	83,784,089	83,786,178	524	59,558.7	7.38	plas: 6, E.R.: 5, cyto: 2
CsP450117	CSS0048711	Chr6	89,527,626	89,529,973	536	61,046.8	6.58	chlo: 9
CsP450118	CSS0012297	Chr6	133,454,516	133,456,890	528	60,222.6	9.51	cyto: 10
CsP450119	CSS0011139	Chr6	163,356,866	163,359,293	542	62,588.1	7.89	plas: 10, E.R.: 3
CsP450120	CSS0005859	Chr6	176,328,928	176,331,843	501	56,884.3	8.14	chlo: 12
CsP450121	CSS0023875	Chr7	11,432,268	11,436,710	506	57,169.6	8.53	chlo: 8, cyto: 2
CsP450122	CSS0012559	Chr7	11,450,735	11,455,490	303	34,739.9	7.54	cyto: 9, cysk: 3
CsP450123	CSS0015881	Chr7	11,526,632	11,530,688	509	57,824.6	7.71	chlo: 8, cyto: 2, E.R.: 2
CsP450124	CSS0025018	Chr7	11,569,462	11,573,506	510	58,294.1	8.62	chlo: 9, E.R.: 2
CsP450125	CSS0029623	Chr7	11,577,262	11,581,724	506	57,241.6	8.53	chlo: 9, E.R.: 2
CsP450126	CSS0000761	Chr7	14,717,745	14,719,058	278	31,579.2	8.12	chlo: 7, mito: 4, nucl: 2
CsP450127	CSS0000063	Chr7	33,218,430	33,220,733	411	46,770.2	9.08	mito: 6.5, chlo_mito: 5.5, chlo: 3.5
CsP450128	CSS0034400	Chr7	33,245,563	33,249,425	518	58,998.2	9.19	chlo: 7, cyto: 2, extr: 2
CsP450129	CSS0037457	Chr7	33,265,170	33,271,135	517	59,508.1	8.85	chlo: 3, nucl: 2.5, cyto: 2, mito: 2
CsP450130	CSS0020807	Chr7	33,298,852	33,303,912	375	42,735.7	8.64	cyto: 10, nucl: 2
CsP450131	CSS0031849	Chr7	33,344,053	33,353,069	346	39,639.4	7.02	cyto: 8, nucl: 3, cysk: 3
CsP450132	CSS0020850	Chr7	33,437,384	33,442,619	418	47,855.9	9.61	cyto: 6, nucl: 3
CsP450133	CSS0031491	Chr7	33,587,239	33,591,611	519	59,155.7	9.19	chlo: 7, cyto: 2
CsP450134	CSS0043900	Chr7	33,613,679	33,615,867	346	39,149.1	8.91	chlo_mito: 5.8, chlo: 5.5, mito: 5
CsP450135	CSS0015144	Chr7	33,795,681	33,799,569	460	52,183.6	8.79	cyto: 8, nucl: 3
CsP450136	CSS0012796	Chr7	34,002,145	34,010,566	526	60,693.7	9.75	plas: 6, E.R.: 5
CsP450137	CSS0040637	Chr7	61,645,359	61,656,955	359	41,404	8.55	extr: 6, cyto: 5
CsP450138	CSS0011272	Chr7	61,693,210	61,704,338	396	44,908	8.57	plas: 9, E.R.: 3, vacu: 2
CsP450139	CSS0017190	Chr7	61,732,332	61,734,620	342	39,174.3	7.71	cyto: 8, nucl: 3
CsP450140	CSS0003581	Chr7	61,766,442	61,769,957	410	46,591.8	8.8	nucl: 4.5, chlo: 4, cyto: 3, cysk_nucl: 3
CsP450141	CSS0008407	Chr7	61,825,320	61,827,469	326	37,267.7	7.2	nucl: 5, cyto: 4, chlo: 2, cysk: 2
CsP450142	CSS0004210	Chr7	63,605,025	63,624,701	545	61,445.7	6.92	chlo: 9, vacu: 2
CsP450143	CSS0021435	Chr7	71,520,249	71,523,168	487	55,413.3	8.05	chlo: 4, nucl: 3.5, cysk_nucl: 2.5
CsP450144	CSS0037928	Chr7	73,641,528	73,664,559	612	68,500.2	6.06	chlo: 9.5, chlo_mito: 7.5, mito: 4.5
CsP450145	CSS0007633	Chr7	74,522,534	74,533,416	523	59,970.3	8.96	vacu: 5, nucl: 2, extr: 2
CsP450146	CSS0047824	Chr7	86,497,691	86,499,217	508	58,299.8	9.06	chlo: 4, nucl: 2, cyto: 2, vacu: 2
CsP450147	CSS0029288	Chr7	117,192,853	117,194,809	339	38,986.1	9.41	plas: 6, vacu: 4, cyto: 2
CsP450148	CSS0042142	Chr7	140,523,844	140,526,080	387	43,847.2	6.88	pero: 9, nucl: 2.5, cyto_nucl: 2.5
CsP450149	CSS0036886	Chr7	141,111,934	141,114,944	514	58,302.3	8.84	pero: 4, E.R.: 3, nucl: 2, plas: 2
CsP450150	CSS0032236	Chr7	156,702,002	156,703,633	340	38,712	8.27	cyto: 7, plas: 2, golg: 2
CsP450151	CSS0049723	Chr7	160,756,496	160,760,365	520	59,887.4	7.12	vacu: 5, plas: 4, E.R.: 3
CsP450152	CSS0036159	Chr7	160,761,801	160,766,232	507	58,201.3	8.72	chlo: 11
CsP450153	CSS0013656	Chr7	160,928,815	160,932,607	497	57,154.6	8.7	plas: 6, E.R.: 3, vacu: 2
CsP450154	CSS0020909	Chr7	160,934,175	160,938,993	507	58,038.1	8.19	chlo: 10, nucl: 3
CsP450155	CSS0050057	Chr7	166,263,270	166,266,119	482	54,468	8.44	vacu: 5, E.R.: 3, golg: 3, extr: 2
CsP450156	CSS0003512	Chr7	170,966,744	170,971,766	517	58,408.4	9.8	chlo: 7, vacu: 3, nucl: 2
CsP450157	CSS0004931	Chr7	171,071,851	171,076,936	517	58,480.4	9.71	chlo: 7, vacu: 3, nucl: 2
CsP450158	CSS0012671	Chr8	43,705,207	43,708,973	479	54,221.6	9.19	chlo: 4, nucl: 3, cyto: 2, extr: 2
CsP450159	CSS0032483	Chr8	53,821,902	53,829,650	475	53,754	9.3	plas: 10, golg: 2
CsP450160	CSS0005915	Chr8	53,989,545	53,996,661	432	49,150	9.05	plas: 7, E.R.: 3, vacu: 2, golg: 2
CsP450161	CSS0012856	Chr8	62,904,952	62,907,370	415	46,956	7.25	cyto: 9, nucl: 3
CsP450162	CSS0018419	Chr8	63,262,295	63,265,026	500	56,067.9	7.91	chlo: 7, nucl: 3
CsP450163	CSS0048887	Chr8	85,834,652	85,838,631	543	61,861.6	8.95	chlo: 11
CsP450164	CSS0049993	Chr8	106,120,350	106,122,209	533	60,670	8.97	chlo: 4, nucl: 3.5, cysk_nucl: 2.5
CsP450165	CSS0031971	Chr8	106,167,056	106,169,021	533	60,686.1	8.84	nucl: 4.5, chlo: 4, cysk_nucl: 3
CsP450166	CSS0037783	Chr8	117,012,922	117,014,382	460	52,954.5	8.22	cysk: 12
CsP450167	CSS0014486	Chr8	118,918,212	118,920,145	384	43,741.9	9.93	chlo: 9, cyto: 2, E.R.: 2
CsP450168	CSS0028162	Chr8	119,656,154	119,657,835	509	56,969.9	8.41	chlo: 4, nucl: 2.5, cyto: 2, plas: 2
CsP450169	CSS0024810	Chr8	119,848,852	119,850,719	347	39,707.1	9.5	cyto: 4, chlo: 3, nucl: 2, pero: 2
CsP450170	CSS0000931	Chr8	127,147,314	127,152,078	523	58,956.4	7.2	chlo: 12
CsP450171	CSS0018950	Chr8	133,390,781	133,392,789	501	57,047.2	7.4	plas: 7, E.R.: 4
CsP450172	CSS0037745	Chr9	56,374,770	56,376,396	451	51,102.9	9.29	chlo: 9
CsP450173	CSS0048509	Chr9	67,166,825	67,171,937	507	57,098	8.7	chlo: 12
CsP450174	CSS0023759	Chr9	67,301,219	67,310,547	503	56,738.6	8.92	chlo: 4, vacu: 3, nucl: 2.5, cyto: 2
CsP450175	CSS0041559	Chr9	67,426,072	67,430,638	405	46,043.1	6.61	cyto: 10
CsP450176	CSS0030922	Chr9	67,450,644	67,454,247	501	57,065	7.96	chlo: 4, vacu: 3, nucl: 2.5, cyto: 2
CsP450177	CSS0040599	Chr9	67,484,285	67,485,987	314	35,366.5	4.93	cyto: 6, nucl: 5
CsP450178	CSS0015107	Chr9	72,397,972	72,400,829	479	54,083.4	8.45	cyto: 4, nucl: 3, plas: 3, chlo: 2
CsP450179	CSS0041517	Chr9	123,141,594	123,149,558	481	55,074.8	7.06	cyto: 10, nucl: 2
CsP450180	CSS0008797	Chr9	123,280,688	123,294,538	516	58,831.5	8.33	plas: 7, E.R.: 4, nucl: 2
CsP450181	CSS0035315	Chr9	125,432,945	125,444,677	532	60,110.8	7.06	chlo: 10
CsP450182	CSS0038702	Chr9	125,883,011	125,888,202	505	56,117.4	8.64	chlo: 12
CsP450183	CSS0036391	Chr9	126,047,814	126,050,906	490	55,086.4	8.48	chlo: 11, mito: 2
CsP450184	CSS0047840	Chr9	126,342,660	126,346,488	505	56,008.3	8.8	chlo: 11
CsP450185	CSS0038388	Chr9	132,881,397	132,900,654	505	57,920.1	10	chlo: 7, vacu: 3, extr: 2
CsP450186	CSS0037893	Chr9	133,005,195	133,031,895	497	57,498.4	9.86	chlo: 9
CsP450187	CSS0037803	Chr9	142,362,180	142,364,280	438	49,712.4	9.34	mito: 5, chlo: 4, cyto: 3, nucl: 2
CsP450188	CSS0013006	Chr10	6,072,656	6,078,938	520	58,826	8.88	chlo: 9, nucl: 2, vacu: 2
CsP450189	CSS0004041	Chr10	87,803,837	87,806,117	495	56,384	9.01	chlo: 10, cyto: 2
CsP450190	CSS0000107	Chr10	96,006,944	96,008,499	268	30,945.6	6.96	cyto: 11
CsP450191	CSS0029119	Chr10	96,199,759	96,204,975	488	55,928.7	9.51	chlo: 4, nucl: 3.5, cysk_nucl: 2.5
CsP450192	CSS0026491	Chr10	96,358,206	96,363,405	488	55,960.8	9.38	chlo: 4, nucl: 3.5, cysk_nucl: 2.5
CsP450193	CSS0006069	Chr10	143,184,466	143,195,402	510	57,883.6	8.04	chlo: 7, nucl: 2, extr: 2, vacu: 2
CsP450194	CSS0003562	Chr10	143,198,486	143,202,344	510	57,880.6	8.53	chlo: 8, nucl: 2, extr: 2, vacu: 2
CsP450195	CSS0004543	Chr11	8,911,509	8,929,571	515	58,041.8	7.07	chlo: 11, vacu: 3
CsP450196	CSS0050131	Chr11	38,183,830	38,189,608	524	59,290.7	8.75	chlo: 11
CsP450197	CSS0036278	Chr11	49,845,009	49,847,411	475	53,828.2	9.47	chlo: 8, extr: 2, vacu: 2
CsP450198	CSS0006273	Chr11	61,621,608	61,623,310	540	61,183.3	8.06	chlo: 10
CsP450199	CSS0007273	Chr11	61,797,869	61,806,173	534	60,463.5	6.98	chlo: 11
CsP450200	CSS0000268	Chr11	65,681,938	65,692,972	464	53,528.3	9.33	chlo: 8, extr: 3
CsP450201	CSS0047867	Chr11	68,877,918	68,879,504	528	60,179.7	9.08	chlo: 13
CsP450202	CSS0022185	Chr11	74,196,638	74,199,420	506	57,400.1	9.15	chlo: 10, cyto: 2
CsP450203	CSS0049182	Chr11	74,277,812	74,280,263	506	57,272.8	9.01	chlo: 11
CsP450204	CSS0022577	Chr11	74,361,840	74,364,239	506	57,146.5	7.98	chlo: 11
CsP450205	CSS0005999	Chr11	74,683,220	74,688,378	505	58,154.3	9.77	plas: 9, E.R.: 3
CsP450206	CSS0002506	Chr11	74,931,080	74,936,724	505	58,170.3	9.77	plas: 9, E.R.: 3
CsP450207	CSS0011714	Chr11	115,241,754	115,251,499	473	52,579.9	8.9	chlo: 11, E.R.: 2
CsP450208	CSS0022212	Chr11	115,906,019	115,911,249	510	57,098.2	9.06	chlo: 4, nucl: 2, cyto: 2, vacu: 2
CsP450209	CSS0030207	Chr12	10,939,273	10,943,334	561	64,840.5	8.14	chlo: 8, nucl: 2.5, vacu: 2
CsP450210	CSS0027743	Chr12	49,026,196	49,030,626	384	43,576	6.87	cyto: 10
CsP450211	CSS0038351	Chr12	54,268,267	54,271,969	511	58,431.5	9.46	chlo: 7, extr: 3, vacu: 2
CsP450212	CSS0022967	Chr12	54,357,672	54,361,305	486	55,420.9	9.12	chlo: 7, extr: 3, vacu: 2
CsP450213	CSS0043926	Chr12	63,481,919	63,487,086	460	52,693.8	9.35	chlo: 8, extr: 2
CsP450214	CSS0032333	Chr12	85,325,766	85,330,502	397	45,110.4	9.35	chlo: 6, vacu: 3, cyto: 2, extr: 2
CsP450215	CSS0018648	Chr12	106,742,782	106,750,553	522	59,544.6	9.94	chlo: 7, extr: 2, vacu: 2
CsP450216	CSS0035056	Chr12	106,845,301	106,859,667	522	59,659.6	9.4	chlo: 7, vacu: 3
CsP450217	CSS0022635	Chr12	107,222,126	107,236,189	522	59,772.8	9.69	chlo: 5, vacu: 3, nucl: 2, extr: 2
CsP450218	CSS0037804	Chr12	118,276,265	118,277,699	398	45,099.6	9.1	vacu: 7, chlo: 2
CsP450219	CSS0021777	Chr12	120,655,778	120,657,940	466	53,129.6	9.52	chlo: 8, cyto: 2, E.R.: 2
CsP450220	CSS0033554	Chr12	124,249,187	124,256,925	506	57,418.7	6.7	chlo: 10
CsP450221	CSS0017388	Chr12	141,134,651	141,138,433	485	54,826.2	8.23	chlo: 5, mito: 3, extr: 2, E.R.: 2
CsP450222	CSS0031994	Chr12	141,143,059	141,144,759	526	59,574.2	9.46	chlo: 13
CsP450223	CSS0020392	Chr12	141,197,699	141,199,398	526	59,628.2	8.72	chlo: 12
CsP450224	CSS0017963	Chr12	141,476,620	141,478,322	506	57,753.6	9.73	chlo: 13
CsP450225	CSS0032565	Chr12	141,479,192	141,480,898	471	53,810.4	9.66	chlo: 14
CsP450226	CSS0006881	Chr12	141,493,120	141,494,819	526	59,322.7	8.62	chlo: 13
CsP450227	CSS0031255	Chr12	141,646,601	141,658,681	535	60,185.2	7.7	extr: 4, chlo: 3, vacu: 3, mito: 2
CsP450228	CSS0043644	Chr12	141,760,261	141,764,010	537	60,837.7	6.92	chlo: 11
CsP450229	CSS0010439	Chr12	143,690,828	143,693,059	494	55,535.1	9.3	chlo: 12, mito: 2
CsP450230	CSS0046908	Chr12	144,412,288	144,414,556	494	55,513.1	8.71	chlo: 12
CsP450231	CSS0046093	Chr12	145,584,715	145,587,065	483	55,449.1	9.24	vacu: 5, golg: 3.5, E.R.: 3, golg_plas: 3
CsP450232	CSS0033566	Chr12	148,837,565	148,839,984	543	61,502.1	8.47	chlo: 11
CsP450233	CSS0044793	Chr12	159,578,061	159,582,685	526	59,093.5	7.33	chlo: 3, E.R.: 3, nucl: 2.5, cyto: 2
CsP450234	CSS0014132	Chr13	36,777,695	36,781,508	450	50,466.5	7.37	chlo: 11, E.R.: 2
CsP450235	CSS0023295	Chr13	41,397,750	41,399,336	511	58,873.4	8.6	chlo: 9
CsP450236	CSS0047471	Chr13	42,535,736	42,540,551	516	58,215.6	6.61	chlo: 11
CsP450237	CSS0041089	Chr13	70,368,832	70,370,526	507	58,278.8	8.05	chlo: 9, nucl: 2.5, cysk_nucl: 2
CsP450238	CSS0000430	Chr13	84,709,899	84,711,452	517	59,085.2	8.93	chlo: 11
CsP450239	CSS0046100	Chr13	84,897,741	84,899,282	498	57,286.2	8.03	chlo: 4, nucl: 2, cyto: 2, plas: 2
CsP450240	CSS0020152	Chr13	84,968,364	84,969,906	497	57,097.9	7.4	chlo: 4, nucl: 2, cyto: 2, plas: 2
CsP450241	CSS0018287	Chr13	85,018,810	85,020,336	508	57,838	8.43	chlo: 13
CsP450242	CSS0006878	Chr13	85,053,137	85,054,663	508	57,811	8.43	chlo: 13
CsP450243	CSS0026636	Chr13	85,209,328	85,210,878	516	58,681.8	9.34	plas: 4, chlo: 3, nucl: 2, cyto: 2
CsP450244	CSS0034482	Chr13	85,292,635	85,294,176	513	58,983	7.42	chlo: 4, nucl: 2, cyto: 2, plas: 2
CsP450245	CSS0007992	Chr13	85,602,192	85,604,051	508	57,902.1	7.42	chlo: 12
CsP450246	CSS0012056	Chr13	85,826,157	85,827,949	514	59,200.3	8.02	cyto: 3, plas: 3, nucl: 2, vacu: 2
CsP450247	CSS0029619	Chr13	86,078,585	86,080,111	508	57,753.9	8.43	chlo: 13
CsP450248	CSS0038256	Chr13	86,127,635	86,129,176	513	58,905.9	7.42	chlo: 4, nucl: 2, cyto: 2, plas: 2
CsP450249	CSS0018099	Chr13	86,278,144	86,290,177	383	43,707.7	5.93	cyto: 12, chlo: 2
CsP450250	CSS0004086	Chr14	9,355,891	9,361,668	499	57,081.4	8.5	chlo: 11, extr: 2
CsP450251	CSS0004195	Chr14	9,722,371	9,723,921	516	59,545.7	7.44	nucl: 4.5, chlo: 4, cysk_nucl: 3
CsP450252	CSS0023665	Chr14	11,317,538	11,320,015	515	59,473	8.51	chlo: 4, nucl: 3.5, cyto: 3
CsP450253	CSS0027183	Chr14	12,279,635	12,282,159	451	52,325.3	6.85	cyto: 10, nucl: 3
CsP450254	CSS0013028	Chr14	12,814,092	12,818,320	479	54,346.5	8.55	chlo: 4, vacu: 3, cyto: 2, extr: 2
CsP450255	CSS0001973	Chr14	12,867,640	12,870,961	491	55,847.3	8.63	chlo: 4, vacu: 3, cyto: 2, extr: 2
CsP450256	CSS0016267	Chr14	47,587,486	47,590,372	520	59,042.2	7.19	chlo: 6, extr: 3
CsP450257	CSS0021413	Chr14	47,865,534	47,869,480	532	60,464.9	8.91	chlo: 10
CsP450258	CSS0002141	Chr14	51,956,895	51,958,816	494	55,683.7	7.84	chlo: 4, nucl: 3.5, cysk_nucl: 2.5
CsP450259	CSS0030825	Chr14	90,565,474	90,567,178	547	61,595.4	6.94	chlo: 13
CsP450260	CSS0030419	Chr14	101,092,487	101,096,739	539	61,583.7	10.17	chlo: 4, nucl: 3.5, cysk_nucl: 2.5
CsP450261	CSS0037030	Chr14	129,518,593	129,530,463	499	57,578.6	9.24	chlo: 4, nucl: 2.5, cyto: 2, vacu: 2
CsP450262	CSS0032858	Chr15	8,921,745	8,925,878	514	58,639.6	7.4	chlo: 14
CsP450263	CSS0014209	Chr15	8,988,231	8,992,273	514	58,639.6	7.4	chlo: 14
CsP450264	CSS0025477	Chr15	13,909,033	13,928,214	472	53,941.2	9.33	chlo: 12
CsP450265	CSS0029697	Chr15	14,274,954	14,282,500	472	53,927.3	9.72	chlo: 10, extr: 3
CsP450266	CSS0016564	Chr15	53,559,524	53,561,095	518	58,788	8.58	nucl: 12, extr: 2
CsP450267	CSS0015933	Chr15	80,694,139	80,709,286	437	49,827.5	7.64	chlo: 9, vacu: 3
CsP450268	CSS0048905	Chr15	95,375,015	95,385,954	518	57,118.4	7.52	E.R.: 5, chlo: 3, vacu: 3
CsP450269	CSS0030176	Chr15	95,611,064	95,621,741	491	54,189.1	8.25	E.R.: 5, vacu: 4, plas: 2
CsP450270	CSS0040139	Chr15	97,937,501	97,940,313	528	58,324	6.98	chlo: 12
CsP450271	CSS0040786	Chr15	106,453,986	106,458,805	511	57,876.1	7.71	chlo: 10
CsP450272	CSS0008715	Chr15	107,239,032	107,244,307	455	51,335.3	7.17	plas: 6, vacu: 3, E.R.: 2
CsP450273	CSS0027949	Chr15	118,532,075	118,554,017	504	58,005.2	9.64	chlo: 9, extr: 2, vacu: 2

### Phylogenetic analysis of CsP450 gene family

**Fig. 1:**
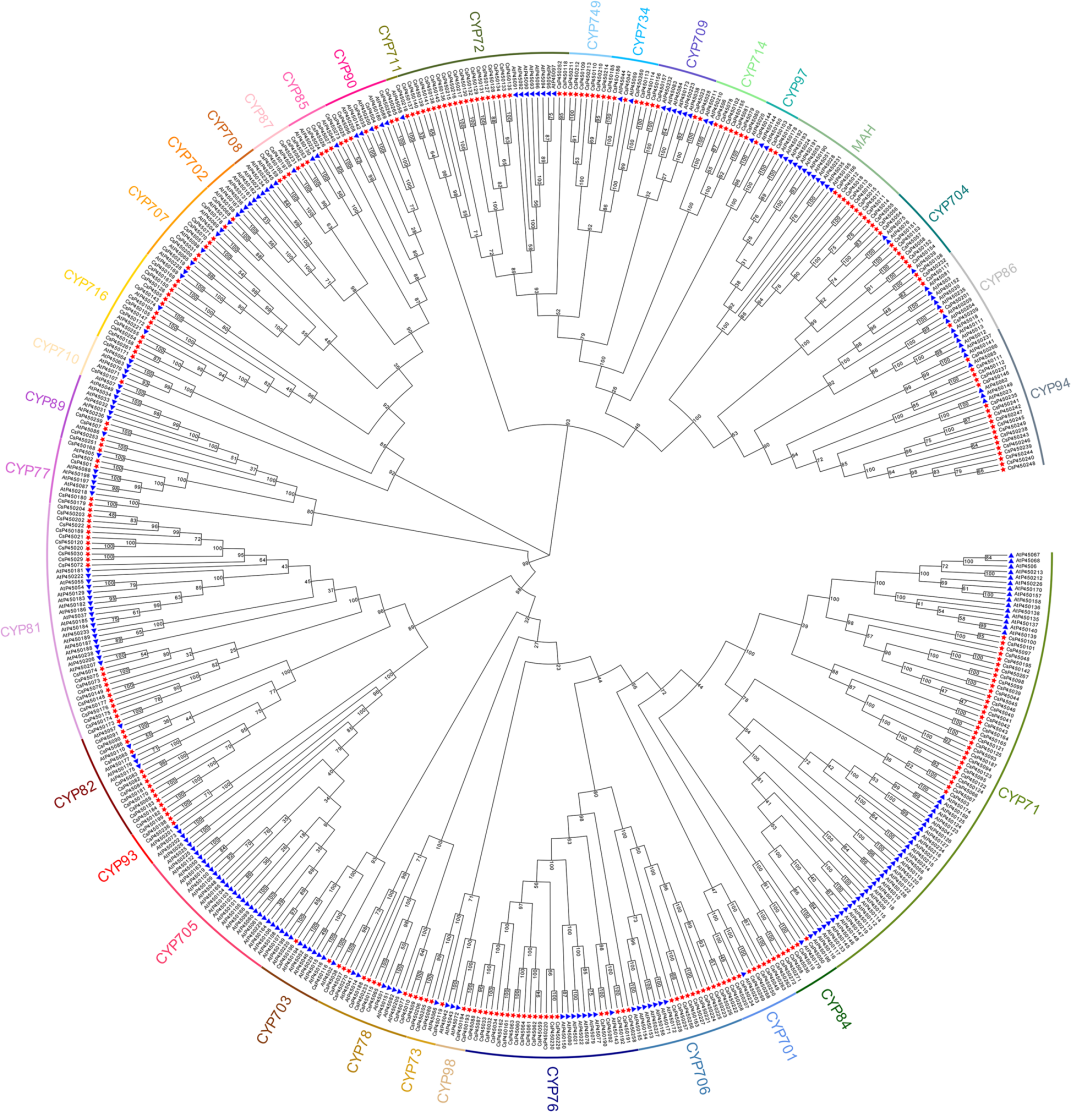
Phylogenetic relationships of *C.sinensis* and *A. thaliana* P450 transporter proteins. The blue triangle represents AtP450 gene and the red asterisk represents CsP450

To gain a deeper understanding of the evolutionary relationships among members of the tea P450 gene family, we conducted multiple sequence alignment of the identified 273 tea P450 proteins with 238 AtP450 protein sequences, followed by cluster analysis to generate a phylogenetic tree (Fig. 1). The results of the phylogenetic tree analysis indicated that the tea P450 proteins belong to 34 subfamilies, including CYP71, CYP72, CYP73, CYP76, CYP77, CYP78, CYP81, CYP82, CYP84, CYP85, CYP86, CYP87, CYP89, CYP90, CYP93, CYP97, CYP98, CYP94, CYP701, CYP702, CYP703, CYP704, CYP705, CYP706, CYP707, CYP708, CYP709, CYP710, CYP711, CYP714, CYP716, CYP734, CYP749, and MAH. The CYP71 subfamily had the most members, containing 31 tea P450 proteins, while the CYP711 subfamily had the fewest members, each containing only one protein. The CYP702, CYP705, and CYP708 subfamilies had no tea P450 proteins, and there were no AtP450 proteins in the CYP749 subfamily. Notably, our evolutionary tree analysis revealed that all subfamilies included tea plant and *A. thaliana* P450 family genes, indicating that the tea plant P450 family shares a common ancestry with the *A. thaliana* P450 family. This analysis provides insights into the evolutionary relationships among tea P450 genes and lays the foundation for further investigations into the functional characteristics of this gene family.

### Gene structure and conserved motif analysis of CsP450s in tea plant

**Fig. 2:**
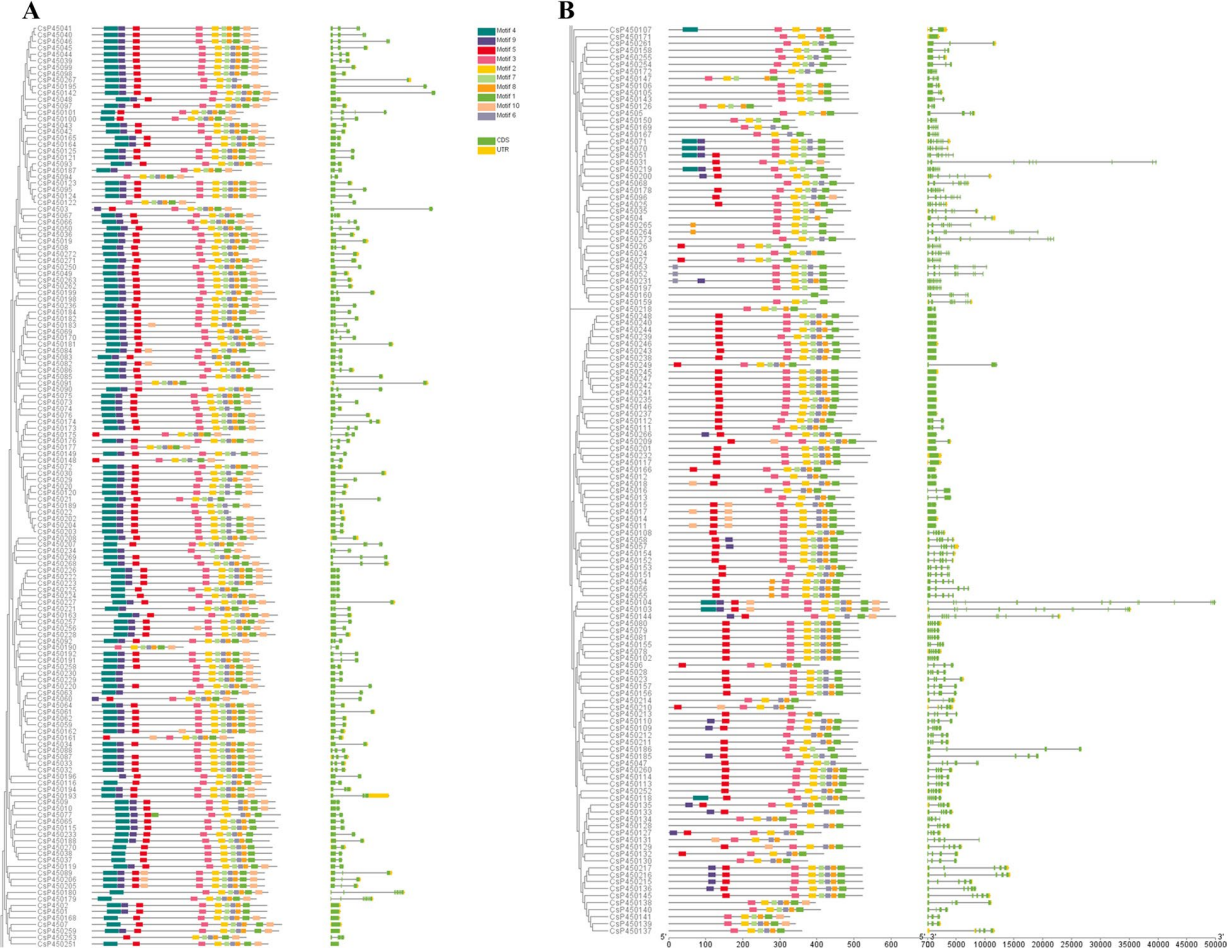
Phylogenetic relationship, gene structure, and distribution of conserved motifs of CsP450 proteins in *C.sinensis*. Diferent motifs are represented by diferent colored numbered boxes

The majority of plant genes are often interrupted by one or more introns or exons. These configurations may be used to investigate the evolutionary link between different members of the respective gene families. Many earlier investigations have observed a correlation between exon/intron distribution patterns and their pertinent biological activities [54]. The evolutionary relationships and gene structures of the tea P450 family members were further investigated by integrating phylogenetic trees, gene structure diagrams, and motif analysis (Fig. 2A and 2B). By using the MEME website, 10 CsP450 proteins' conserved motifs were identified. The analysis revealed that the number of exons in tea P450 family genes ranged from 1 to 14, with 27 genes lacking introns and only one exon. In addition, ten conserved motifs (Motif1-Motif10) were identified in the CsP450 family proteins (Figure S1). The number of conserved motifs in tea P450 family genes varied from 1 to 10, with Motif5 to Motif8 being the most frequently occurring motifs in all genes. Furthermore, there were significant differences in the patterns of conserved motifs and gene structures between type A and non-type A P450s. For example, type A includes the CYP71 clan, which contains the following sub-families: CYP71, CYP78, CYP82, CYP89 and CYP736, while the non-type A clan included all CYPs other than CYP70 types. However, similar patterns were observed within the same subfamily, which enhanced the credibility of the phylogenetic relationships and population classifications.

### Analysis of cis-acting elements of CsP450 gene family

Cis regulatory elements (CREs) are a family of non-coding DNA components that regulate gene expression at various developmental stages by influencing the transcription of nearby genes [55]. To investigate the potential response of CsP450 family members to growth and development, stress and other environmental cues, the promoter regions were analyzed using PlantCARE. The results showed that the major cis-acting elements included abscisic acid responsive elements (ABRE), jasmonic acid response elements (CGTCA-motif), low temperature responsive element (LTR), MYB binding site involved in drought-inducibility (MBS), gibberellin-responsive regulatory element (TATC-box), salicylic acid responsive element (TCA-element) and auxin-responsive element (TGA-element) (Figure S2). The predicted results further suggest that the tea CsP450 family plays an important role in regulating growth and development processes, hormone signal transduction, and response to environmental stress.

### Chromosomal distribution analysis of CsP450s in tea plant

**Fig. 3:**
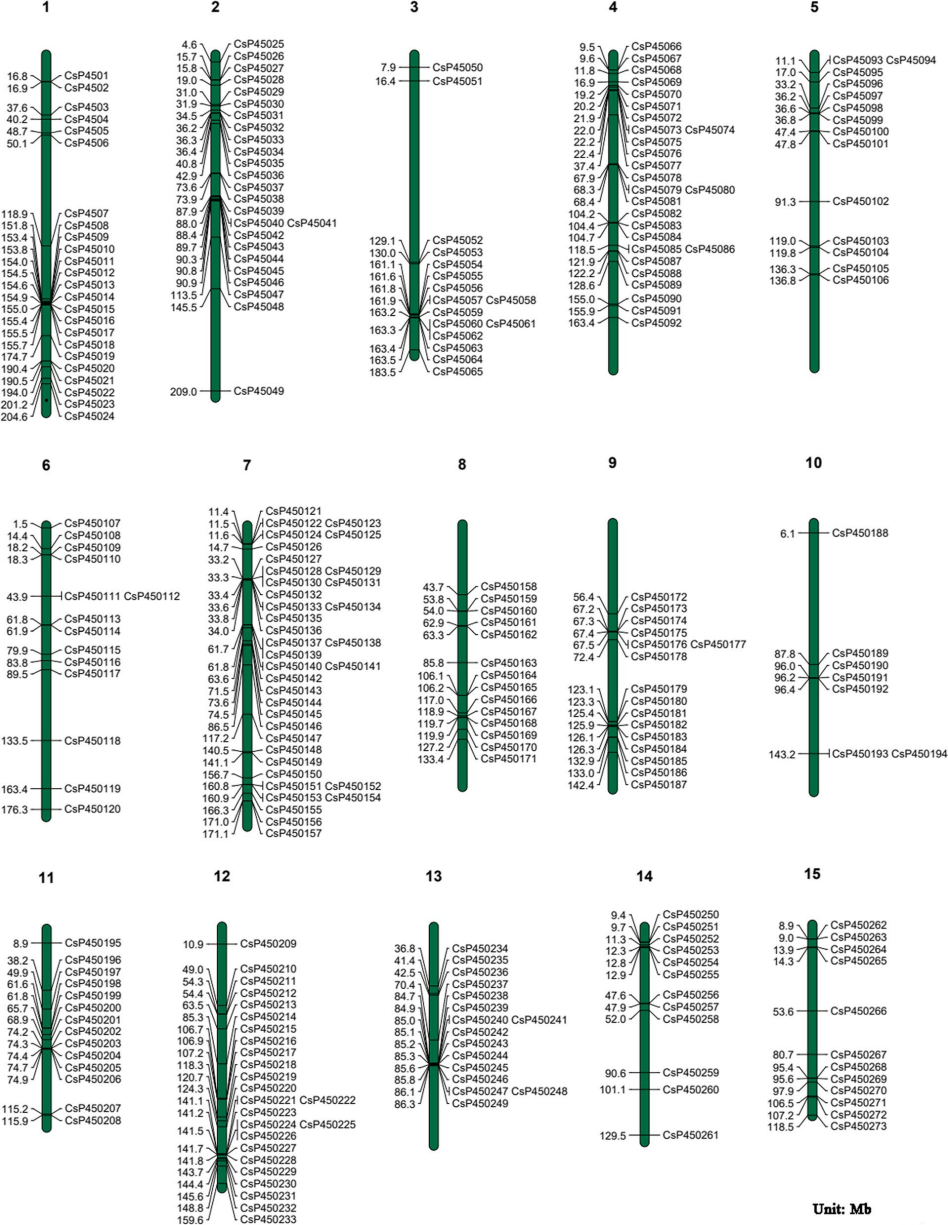
Chromosomal locations of P450 transporter proteins in *C.sinensis*. Chromosome numbers are represented at the top of each chromosome

Based on the genome annotation of the tea plant, we investigated the physical locations of CsP450s on tea plant chromosomes, and the results are presented in Fig. 3. The chromosome localization results of P450 genes in tea plants showed that all 15 chromosomes of tea plants contain P450 genes, indicating that the chromosome distribution of P450 genes in tea plants is biased. Among them, chromosomes 1, 2, 4, 7, and 12 have the most P450 genes, while chromosome 10 has the fewest. In addition, it was found that some CsP450 genes are closely linked, and 37 pairs of genes exhibit gene tandem duplication.

### Gene duplication relationship and collinearity analysis of CsP450 genes

**Fig. 4:**
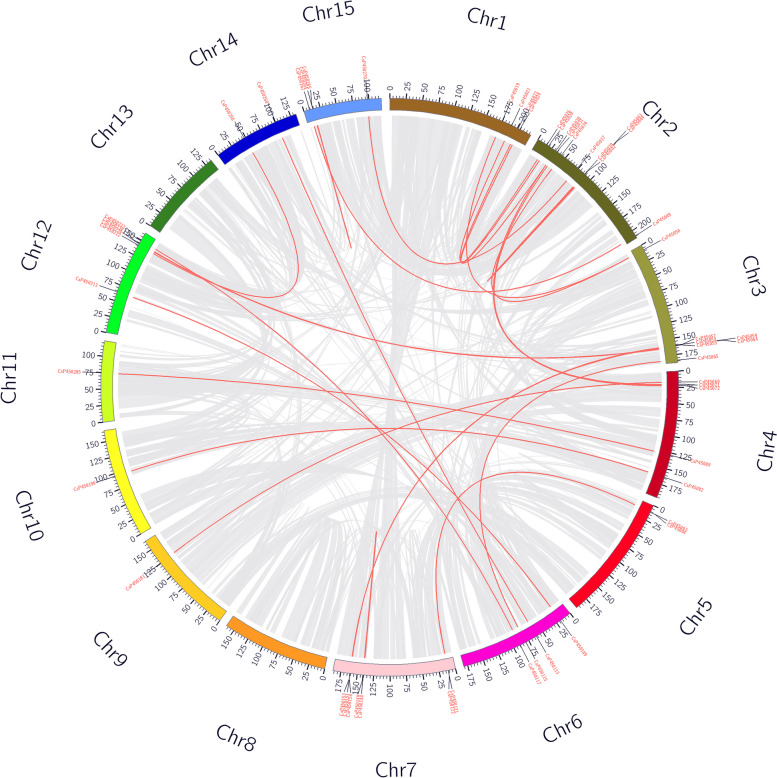
Synteny analysis of P450 genes in *C.sinensis*. The red line is a large segment replication between gene family members

The investigation of gene duplication and amplification is crucial for exploring the evolution and expansion of the P450 gene families in tea plant. To investigate gene duplication events in the CsP450 gene family of tea plants, the MCScanX algorithm was used to analyze collinearity and gene duplication in the tea plant genome. Gene duplication and amplification between P450 genes provide important evidence for studying the evolution and expansion of gene families. Red lines linking two chromosomal parts represent syntenic regions. Analysis of large-scale gene duplication within the P450 gene family revealed that 37 pairs of genes participated in tandem duplication and 28 pairs of genes were collinear, providing the driving force for the evolution of tea plants (Fig. 4). In addition, duplication was most frequent in chromosomes 2 and 3, which is also the main reason for the higher number of *CsP450* genes on these chromosomes. According to the aforementioned findings, tandem duplication and segmental duplication both contributed to the growth of the CsP450 family, although the former had a more significant impact.

**Fig. 5:**
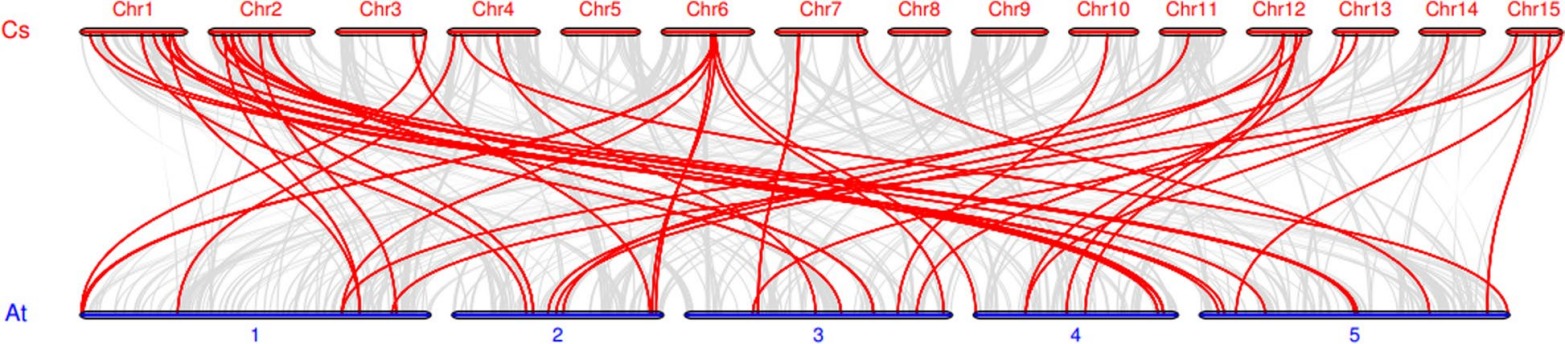
Synteny analysis of P450 genes between the genomes of *C.sinensis* and *A. thaliana*. Cs represents *C. sinensis* genome and AT represents *A. thaliana* genome. The gray lines are all collinear relationships among different genomes, and the colored lines are collinear relationships among P450 gene family genes

Family members of a gene family often evolve from a single ancestral gene. Therefore, using collinearity analysis to study the relationship between P450 gene families in tea plants and *A. thaliana* genomes helps to understand the origin and evolutionary relationship of P450 genes (Fig. 5). The results showed that 41 homologous P450 genes were co-constructed in tea plants and *A. thaliana*, with more homologous P450 genes found in chromosomes 1 and 2 of tea plants, while no homologous P450 genes were found on chromosomes 5, 8, and 9. In addition, multiple tea plant P450 genes were identified as homologous to a single AtP450 gene, and multiple *AtP450* genes were also homologous to a single tea plant P450 gene. This collinearity relationship suggests that the expansion of this gene family may have occurred before the divergence of tea plants and *A. thaliana*.

### Selection pressure analysis of CsP450 genes

Throughout the course of evolution, gene duplication events often lead to the divergence of duplicated genes from their initial specialized functions. This divergence may manifest as non-functionalization, sub-functionalization, or neo-functionalization [56]. We calculated Ka/Ks values from inter and intra genomic/subgenomic combinations of the tea plant in order to study the influence of Darwinian positive selection and the magnitude of selection pressure on divergence of P450 duplicated genes. As the majority of the Ka/Ks values were less than 1, it was assumed that after segmental and whole genome duplication, the CsP450 gene family had undergone strong purifying selection pressure with limited functional divergence (Table S1).

### Protein–protein interaction network analysis of CsP450 genes

**Fig. 6:**
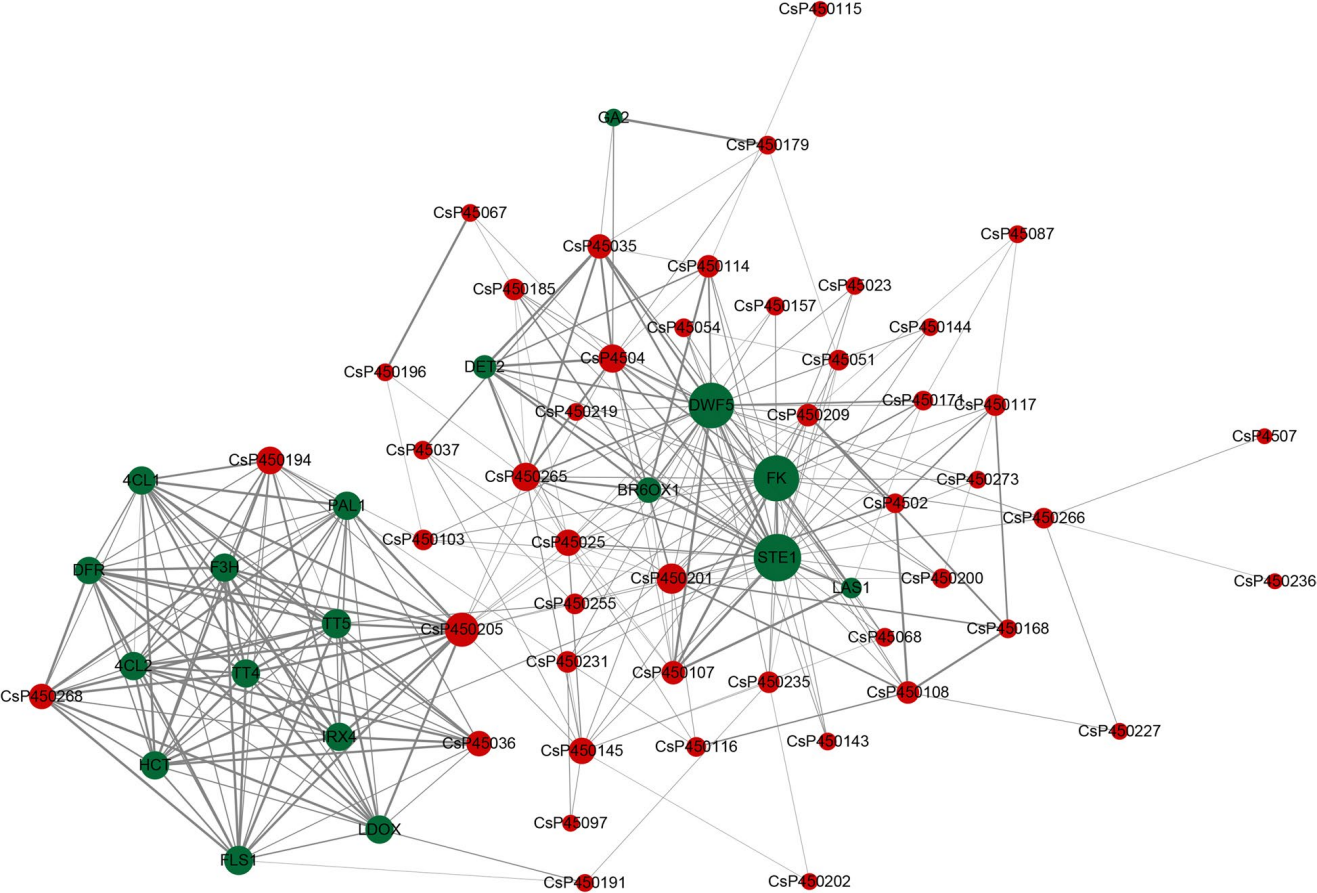
Protein interactions network diagram of P450 genes in *C.sinensis*. The red dots are CsP450 genes; the green dots are other genes added based on the String database. The size of the dots represents the size of the degree, and the thickness of the line represents the level of confidence

Analysis of protein–protein interactions (PPI) is a crucial way to understand protein function. Using the protein interaction network of *Arabidopsis*, we mapped and analyzed the protein interaction network of tea P450 proteins (Fig. 6). The results showed that 317 interactions were detected to be involved in the PPI network. The protein interaction map showed that multiple tea P450 genes have interacting target proteins, such as phenylalanine ammonia-lyase PAL1, flavonoid synthesis gene F3H, brassinosteroid synthesis pathway genes DWF5, DET2, STE1, and BR6OX1, among others. Additionally, there may also be protein–protein interaction relationships between tea P450 proteins, such as CsP450107, CsP450108, CsP450116, CsP450145, CsP450231, and CsP450266, among others. Therefore, the protein interaction network analysis further supports the hypothesis that tea P450 proteins may participate in multiple physiological pathways through protein interactions.

### Tissue-specific expression of CsP450 genes

**Fig. 7:**
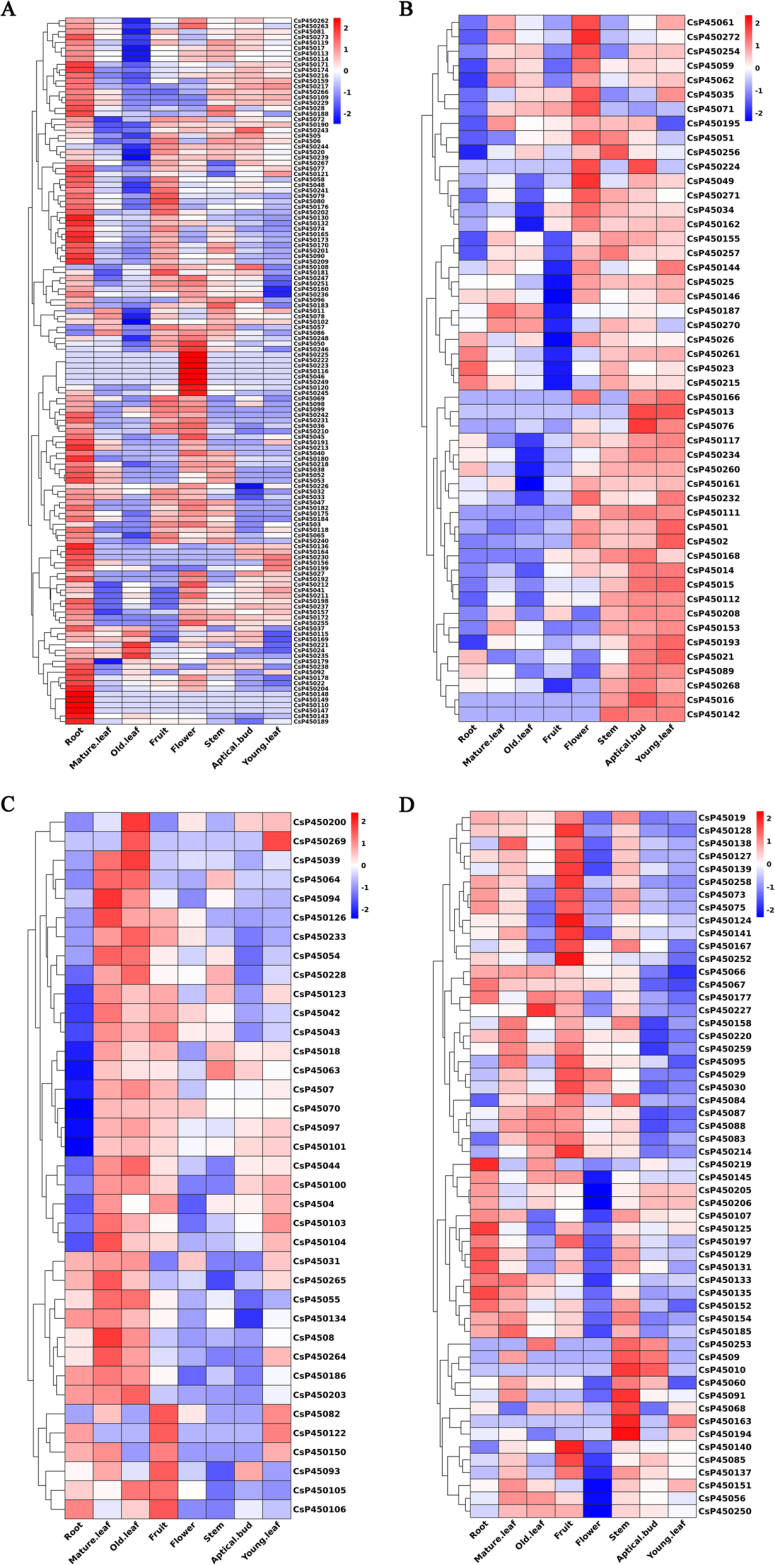
Heatmap of CsP450 genes expression clustering in eight different tissues in *C.sinensis*. The heat blocks represent high and low expression, with red color representing high gene expression and blue color representing low gene expression

Understanding the tissue-specific expression patterns of genes is crucial for elucidating their roles in plant growth, development, and responses to environmental stresses [57]. The expression patterns of genes in different tissues are closely related to their biological functions. In this study, we analyzed RNA-Seq data from eight different tissues of tea plants (apical buds, flowers, fruits, young leaves, mature leaves, old leaves, roots, and stems) to analyze the tissue-specific expression profiles of the P450 gene family. Normalized FPKM expression values were used to construct a digital expression profile heatmap. The CsP450s exhibited a diverse expression pattern. The results showed that the P450 gene family had high expression levels in the roots and stems, while their expression levels were low in mature and old leaves in tea plants (Fig. 7–1, -2). The clustering results indicated that P450 genes in the same subfamily exhibited similar expression patterns.

### Expression analysis of CsP450s in response to drought and cold stress

**Fig. 8:**
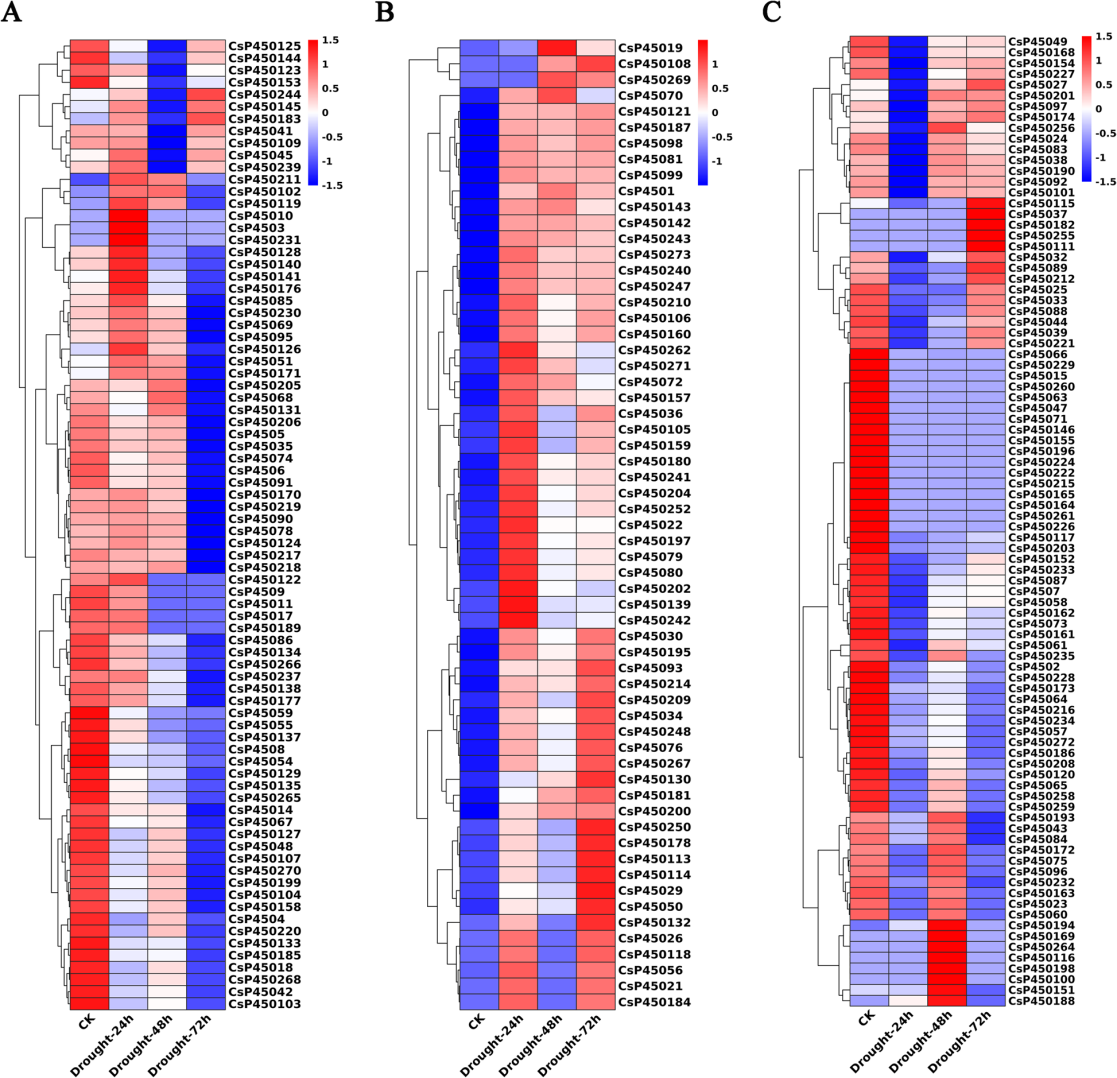
Heatmap of CsP450 genes expression clustering under drought stress in *C. sinensis*. The heat blocks represent high and low expression, with red color representing high gene expression and blue color representing low gene expression

**Fig. 9:**
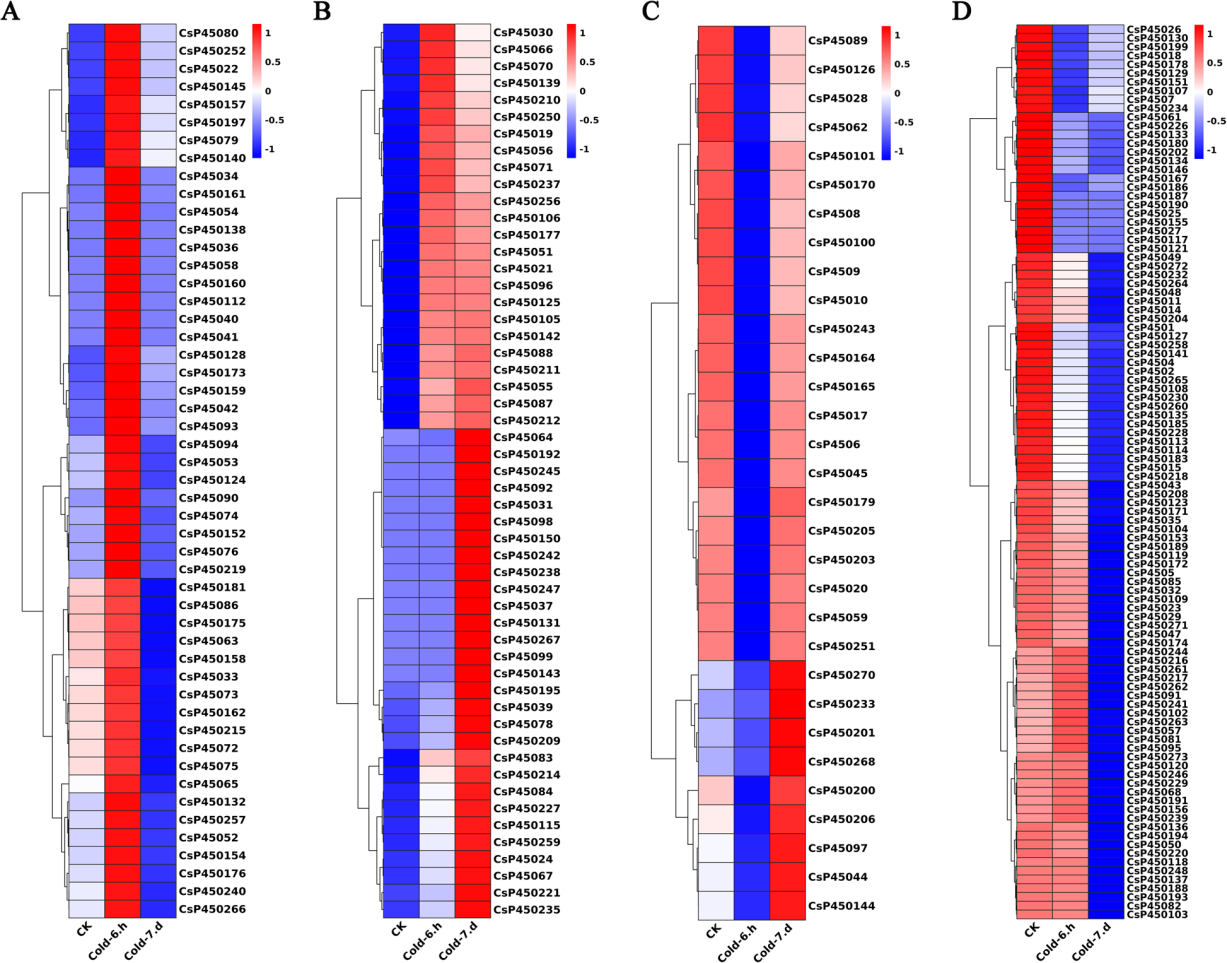
Heatmap of CsP450 genes expression clustering under cold stress in *C. sinensis*. The heat blocks represent high and low expression, with red color representing high gene expression and blue color representing low gene expression

To investigate the response of the P450 gene family to drought and cold stress in tea plants, transcriptome sequencing data from tea plants subjected to PEG treatment (24 h, 48 h, and 72 h) and cold stress (6 h and 7 d) were analyzed. The results indicated that the expression of CsP450 genes in response to drought stress followed one of three trends: initial upregulation followed by downregulation, sustained upregulation, or continuous downregulation (Fig. 8). Similar expression patterns were also observed under cold stress (Fig. 9). Furthermore, the clustering analysis of the CsP450 gene family revealed that genes from the same subfamily displayed similar expression patterns. These findings demonstrate that the expression of the CsP450 gene family is modulated in response to drought and cold stress in tea plants. These results may provide valuable insights into the molecular mechanisms underlying stress tolerance in tea plants and could facilitate the development of stress-resistant tea cultivars in the future.

### Expression analysis of CsP450s in response to drought and cold stress

**Fig. 10:**
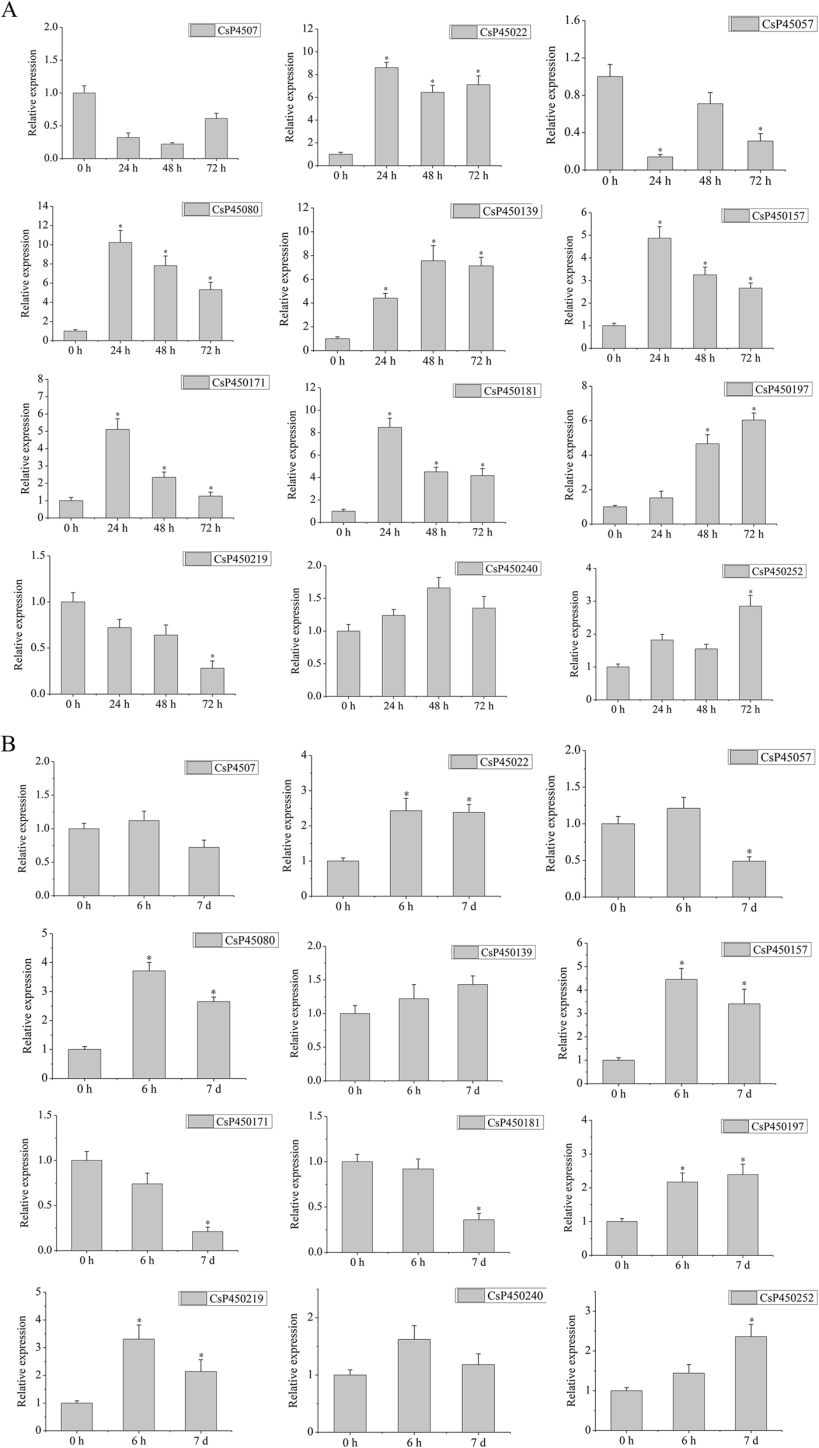
The relative expression levels of selected CsP450 genes under drought and cold treatments, as determined by qPCR. **A** The expression profiles of genes under drought treatments at different time points. **B** The expression profiles of genes under cold treatments at different time points. Error bars show standard deviations among three independent biological replications. * represents *p* < 0.05

CsP450 genes are essential in tea plant response to environmental abiotic and biotic stresses. To further validate the expression patterns of the selected CsP450 genes under drought and cold stress, a quantitative real-time polymerase chain reaction (qPCR) was performed on 12 different CsP450 genes. The results indicated that the qPCR data were generally consistent with the transcriptomic data (Fig. 10). Specifically, under drought stress, CsP450139, CsP450197, and CsP450252 exhibited a continuous upregulation trend, with an approximately 8-, 5- and threefold increase, while CsP450219 showed a continuous downregulation trend compared with control. Besides, CsP45080, CsP450157 and CsP450181 showed an initial upregulation followed by a downregulation trend (Fig. 10A). However, the transcript level on each time points of CsP450240 showed no significant difference than control, with the maximum relative expression reaches 1.6 times at 48 h.

Under cold stress, CsP45080, CsP450157 and CsP450219 exhibited an increase followed by a decrease in expression levels, while CsP45022, CsP450197 and CsP450252 showed a continuous upregulation trend compared with control, with an approximately 2.5-, 2.4- and 2.3-fold increase. Conversely, CsP450171 and CsP450181 showed a continuous downregulation trend compared with control (Fig. 10B). Besides, the transcript level on each time points of CsP4507, CsP450139, and CsP450240 showed no significant difference than control. The findings from the qPCR analysis support the expression patterns observed in the transcriptomic data, thereby providing further evidence for the involvement of CsP450 genes in response to drought and cold stress in tea plants.

## Discussion

The cytochrome P450 genes catalyze various reactions, including growth, development, and biosynthesis of secondary metabolites [13]. Gene identification and functional classification are essential for studying the function of gene families. As an important supergene family, cytochrome P450s have been identified at the genome level with the availabilities of the whole genome sequence in various plants. However, little is known about how these P450 genes respond to biotic and abiotic stresses and how they participate in the growth and development of tea plants. In this study, 273 non-redundant P450 genes were identified from the tea plant genome, and these genes are similar to those found in *Arabidopsis*. Then, a comprehensive study was conducted on the phylogenetic relationships, conserved motifs, gene structures, gene duplication events, cis-acting elements, and gene expression patterns in different tissues of tea plant members of this gene family. Besides, we analyzed the expression profile from RNA-Seq data related to drought and cold stress. The study contributes detailed knowledge on the CsP450 gene family and will help in comprehending the functional divergence of P450 genes in tea plants.

Recent genome sequencing revealed an approximate 3.0 Gb genome size for two representative elite tea plant cultivars [58]. The phylogenetic tree topology of tea plant and *Arabidopsis* P450s showed similar clustering, indicating a certain degree of conservation of the P450 multi-gene family in plants. In the current phylogenetic classification of plant P450s, the plant P450 family is divided into nine different subfamilies, including CYP51, CYP71, CYP710, CYP711, CYP72, CYP74, CYP85, CYP86, and CYP97 subfamilies [11]. Among the subfamilies present in the tea are CYP710, CYP711, CYP71, CYP72, CYP74, CYP85, CYP86, and CYP97. Many plant-specific enzymes encoded by P450 genes play a role in the metabolism of secondary products, belonging to the largest subfamily, CYP71, which has the most members in tea plants. The CYP71 subfamily is classified as type A P450s, and the remaining eight subfamilies are classified as non-A type [59]. Most type A genes encode plant-specific enzymes that act on the metabolism of secondary products (such as phenylpropanoids and alkaloids), while non-A type genes are mainly involved in the synthesis of hormones and other compounds [60]. These analyses provide critical information for studying the phylogeny of the cytochrome P450 gene family.

A recent study found that multiple cytochrome P450 (P450) genes induced by both biotic and abiotic stressors contain recognition sites for MYB and MYC transcription factors, ACGT core sequences, TGA-boxes, and W-boxes for WRKY transcription factors [61]. These cis-acting elements are known to be involved in the regulation of plant defense, and the response of each P450 gene to various stressors is strictly controlled [17]. In this study, numerous hormone-induced regulatory elements, such as TATC-box, TCA-element and TGA-element, and cis-acting elements involved in responses to abiotic stress, such as low temperature and drought, were identified in the promoter sequences of tea plant P450 genes.

Although the functions of multiple subfamilies of the P450 family have been extensively explored, the molecular basis for the transcriptional activation of many P450 genes by receptor-mediated signaling remains in its early stages [62]. Furthermore, it should be noted that subcellular localization of some P450 enzymes, some of which may have more than one organelle localization, such as CsP45052 may function in the plasma membrane, mitochondrial membrane or endoplasmic reticulum. In particular, many P450-catalyzed reactions in plants may produce toxic compounds if released into the cytoplasm [63].

The evolution of organisms is mostly fueled by gene duplication. Tandem duplication (TD) and segmental or whole-genome duplications (S/WGD) are the two basic mechanisms by which gene duplication has taken place [63]. In our study, segmental duplication of 28 P450 gene pairs was found in the tea plant. It was assumed that the ancient triplication WGD throughout evolution was responsible for these genes. Together with the segmental duplication events, 37 tandem duplication events were found, suggesting that tandem duplication played a major role in the proliferation of P450 genes in tea plants. These results were in line with the phenomenon observed in citrus and grapevine, where the majority of CYP genes were created through tandem duplication [64, 65].

Previous studies have revealed that plant P450 plays significant roles in different kinds of biochemical pathways and plays important roles in multiple biological processes, including development and stress response [66, 67]. The phenylpropanoid (PPP) pathway was discovered in the CsP450 PPI network, a crucial secondary metabolism pathway implicated in numerous biosyntheses, including the formation of lignin, radical scavenging, signalling molecules, and reproduction. In our study, the CsP450 genes' expression profiles were examined during various developmental stages as well as in response to drought and cold stresses. The findings suggested that the CsP450 genes could be grouped into various groups based on their expression patterns, and the genes within each cluster might be involved in a number of related functions. Furthermore, additional research is necessary to uncover the specific roles of individual CsP450 genes in the stress response and to assess their potential for the genetic improvement of tea plants.

## Conclusions

In this study, we identified a total of 273 CsP450s family genes in the tea plant genome, which can be divided into A and non-A types, consisting of 34 subfamilies. We analyzed their structures and functions and found that subfamilies within the same type have similar exon–intron structures and motif compositions. In addition, we identified some cis-acting elements related to secondary metabolism and stress response. The results of collinearity and synteny suggested that the WGD/segmental duplications might mainly contribute to the expansion of the P450 gene family during evolution. Furthermore, our findings suggest that the CsP450 gene family is implicated in the response of tea plants to drought and cold stress. These results offer novel insights into the molecular mechanisms that underlie stress responses in tea plants and could have practical implications for breeding stress-tolerant tea cultivars.

### Supplementary Information

**Additional file 1.**

**Additional file 2.**

**Additional file 3.**

**Additional file 4.**

**Additional file 5.**

**Additional file 6.**

## Data Availability

The datasets generated and/or analysed during the current study are available in the GenBank repository [2696664].
