# Supplementary figures and images for: Genome-wide analysis of the P450 gene family in tea plant (Camellia sinensis) reveals functional diversity in abiotic stress
Source: BMC Genomics. 2023 Sep 11;24:535. doi: 10.1186/s12864-023-09619-4 (PMC10494425; doi:10.1186/s12864-023-09619-4)

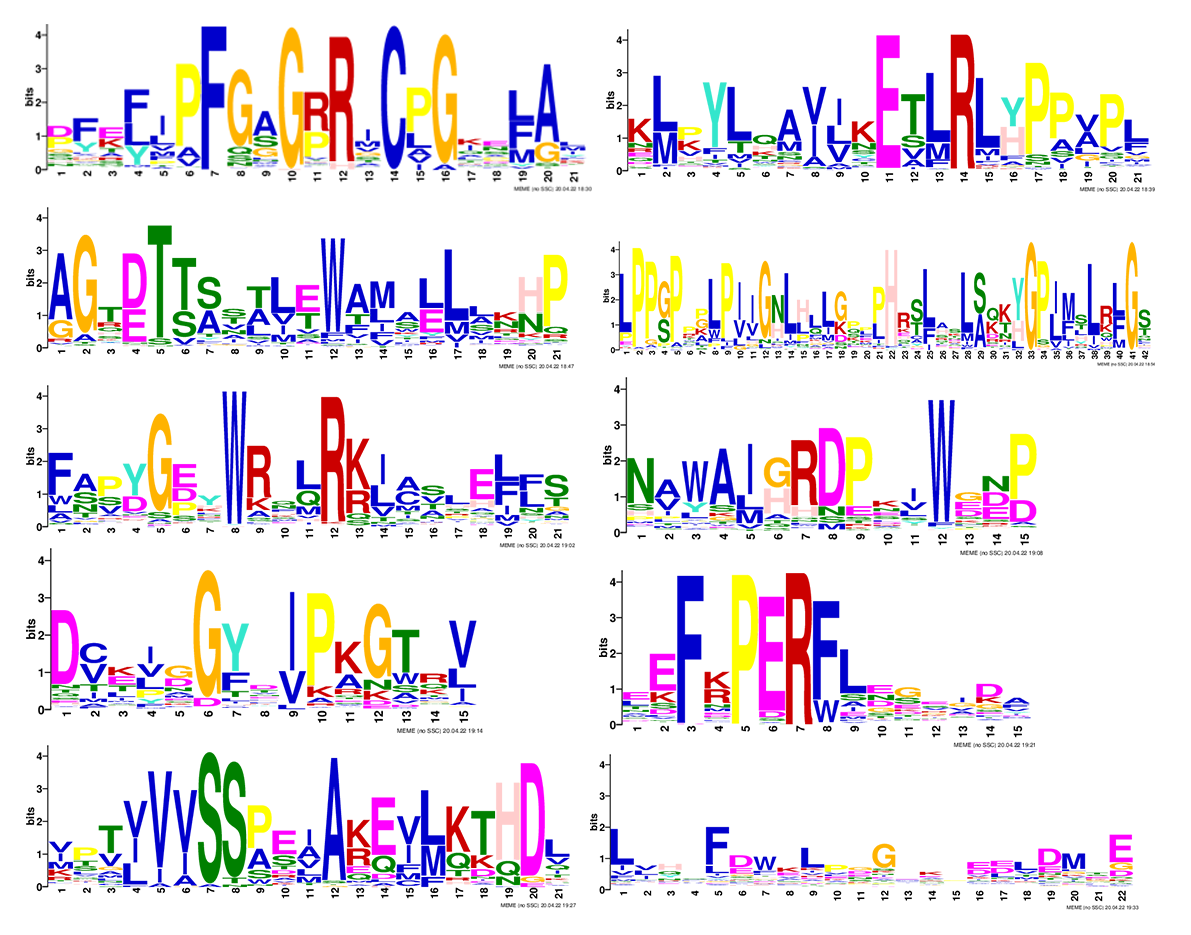

Supplement: Supplementary file 1 — Additional file 1. [file 12864_2023_9619_MOESM1_ESM.tif]

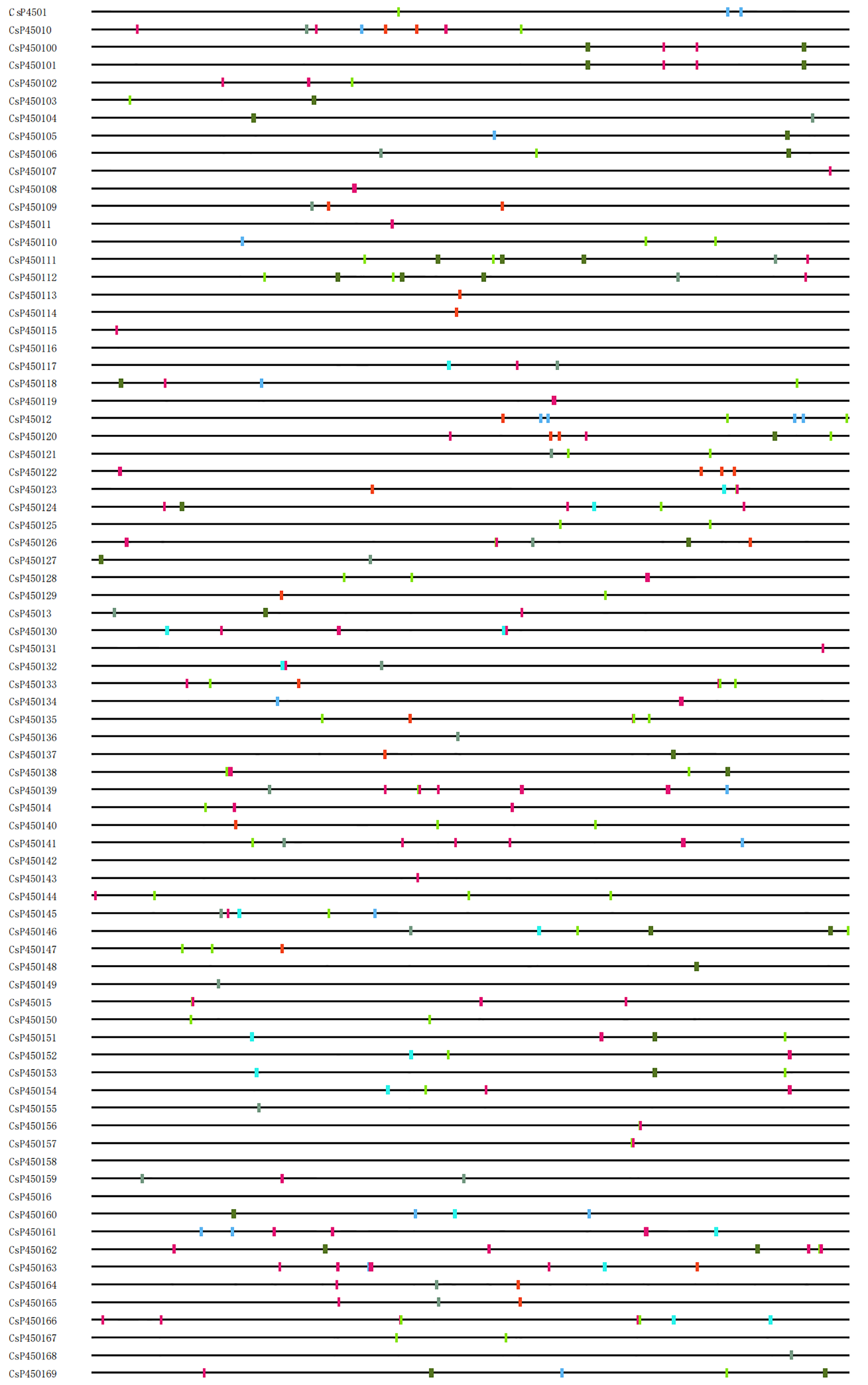

Supplement: Supplementary file 2 — Additional file 2. [file 12864_2023_9619_MOESM2_ESM.tif]

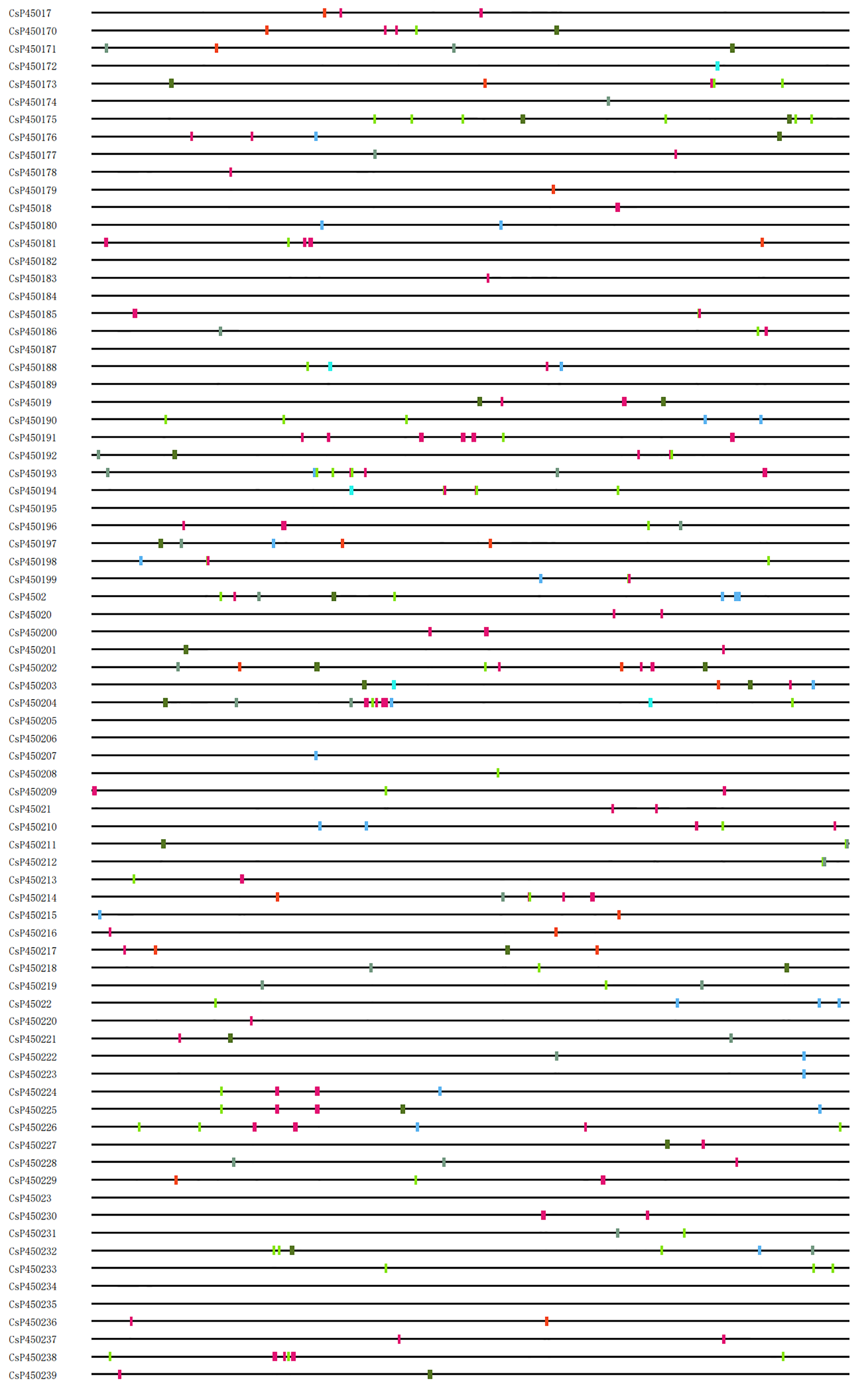

Supplement: Supplementary file 3 — Additional file 3. [file 12864_2023_9619_MOESM3_ESM.tif]

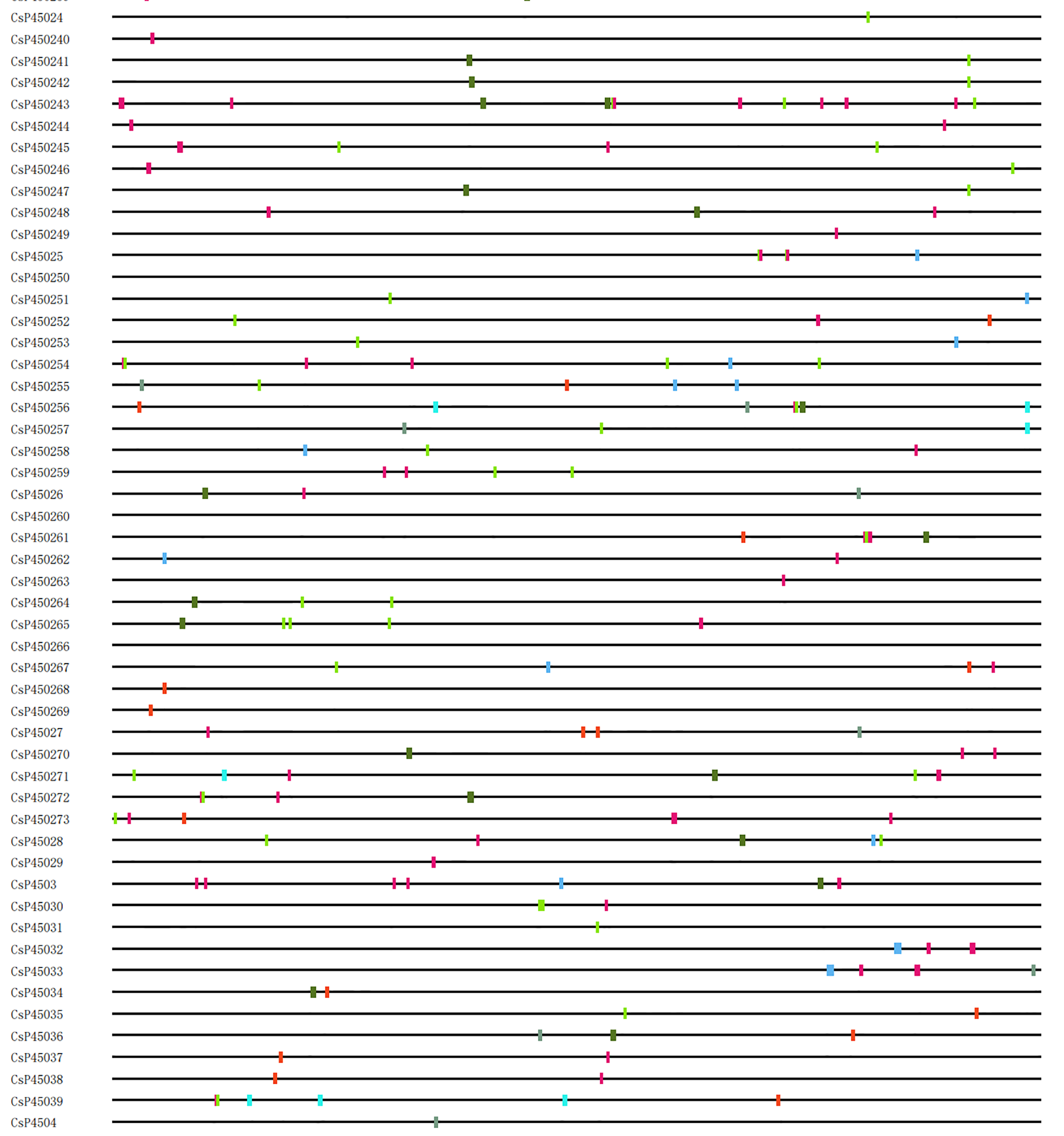

Supplement: Supplementary file 4 — Additional file 4. [file 12864_2023_9619_MOESM4_ESM.tif]

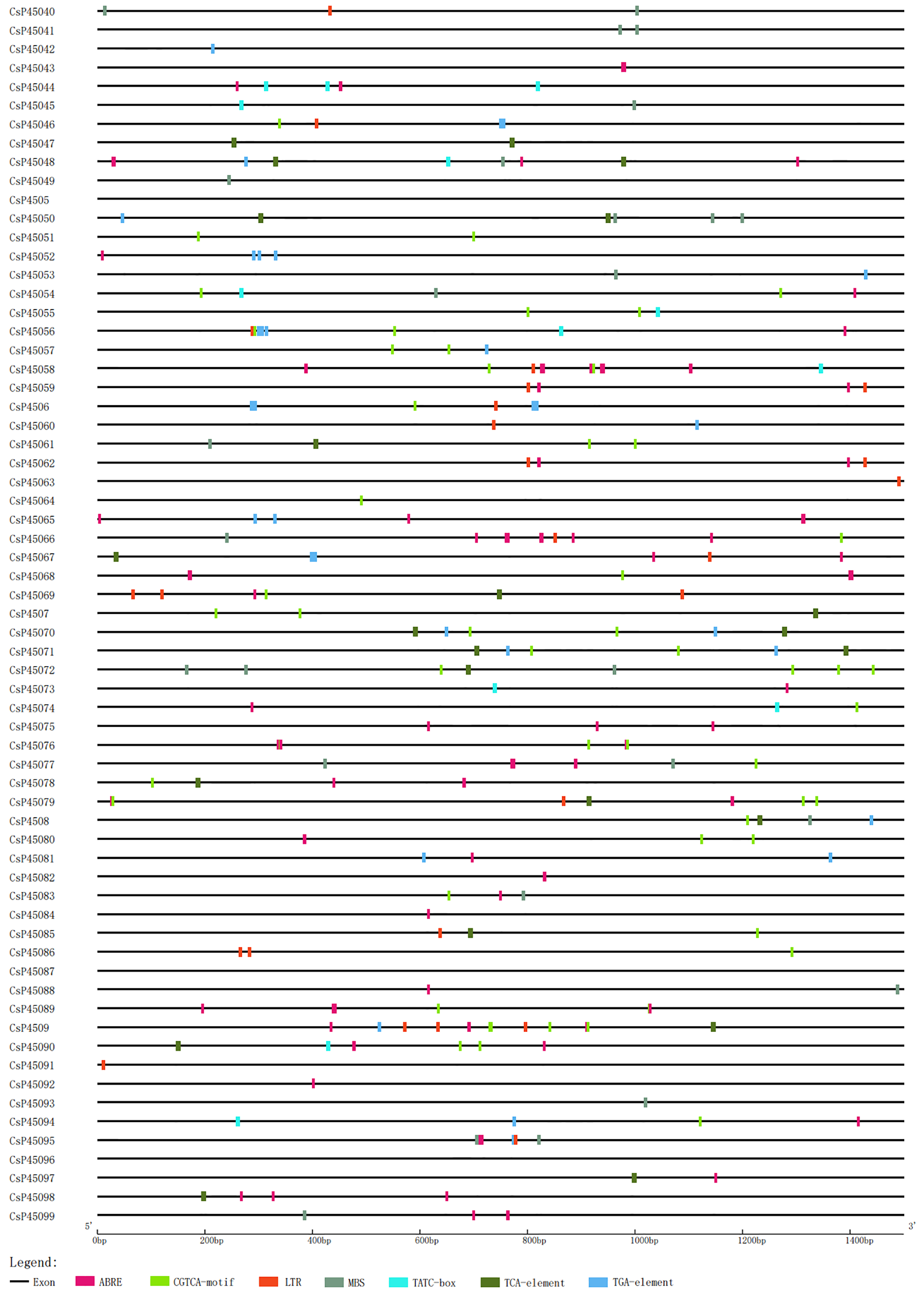

Supplement: Supplementary file 5 — Additional file 5. [file 12864_2023_9619_MOESM5_ESM.tif]
